# Modelling confounding effects from extracerebral contamination and systemic factors on functional near-infrared spectroscopy

**DOI:** 10.1016/j.neuroimage.2016.08.058

**Published:** 2016-12

**Authors:** Matthew Caldwell, Felix Scholkmann, Ursula Wolf, Martin Wolf, Clare Elwell, Ilias Tachtsidis

**Affiliations:** a University College London, Department of Medical Physics and Biomedical Engineering, Biomedical Optics Research Laboratory, Gower Street, London WC1E 6BT, United Kingdom; b University Hospital Zurich, Department of Neonatology, Biomedical Optics Research Laboratory, 8091 Zurich, Switzerland; c University of Bern, Institute of Complementary Medicine, 3012 Bern, Switzerland

**Keywords:** Functional near-infrared spectroscopy, Brain, Modelling, Confounding, Scalp, CO2 reactivity

## Abstract

Haemodynamics-based neuroimaging is widely used to study brain function. Regional blood flow changes characteristic of neurovascular coupling provide an important marker of neuronal activation. However, changes in systemic physiological parameters such as blood pressure and concentration of ${\mathrm{CO}}_{2}$ can also affect regional blood flow and may confound haemodynamics-based neuroimaging. Measurements with functional near-infrared spectroscopy (fNIRS) may additionally be confounded by blood flow and oxygenation changes in extracerebral tissue layers. Here we investigate these confounds using an extended version of an existing computational model of cerebral physiology, ‘BrainSignals’. Our results show that confounding from systemic physiological factors is able to produce misleading haemodynamic responses in both positive and negative directions. By applying the model to data from previous fNIRS studies, we demonstrate that such potentially deceptive responses can indeed occur in at least some experimental scenarios. It is therefore important to record the major potential confounders in the course of fNIRS experiments. Our model may then allow the observed behaviour to be attributed among the potential causes and hence reduce identification errors.

## Highlights

• Confounding of fNIRS haemoglobin signals is simulated using a computational model.
• Model is extended to simulate scalp haemodynamics.
• Changes in blood pressure and CO_2_ can mimic and mask functional activation.
• Experimental recording of systemic factors is recommended to aid interpretation.

## 1 Introduction

Neuroimaging techniques relying on changes in tissue haemodynamics and oxygenation, such as functional near-infrared spectroscopy (fNIRS) and blood oxygen level dependent (BOLD) based functional magnetic resonance imaging (fMRI), have been widely and productively used to investigate cerebral function. Regional haemodynamic changes provide a marker of neuronal activation due to tight neurovascular coupling (Logothetis et al., 2001, Gagnon et al., 2015, Weber, 2015).

It is well known that a variety of systemic physiological factors also significantly affect cerebral blood flow (Rostrup et al., 2002, Ainslie and Duffin, 2009, Battisti-Charbonney et al., 2011, Sobczyk et al., 2016). Changes to these factors can occur in the course of functional experiments. Such changes may, of course, be unrelated to the experimental procedure, but may also arise more systematically (Tachtsidis and Scholkmann, 2016). For example, task-evoked changes in mean blood pressure have been demonstrated in protocols including anagram solving (Tachtsidis et al., 2009), visual stimulation (Minati et al., 2009) and video gaming (Tachtsidis and Papaioannou, 2013). Similarly, changes to blood ${\mathrm{CO}}_{2}$ concentration have been observed in tasks involving speaking (Scholkmann et al., 2013a) and mental arithmetic (Scholkmann et al., 2013b). In the case of fNIRS, there is further scope for confounds arising from haemodynamic/oxygenation changes in the extracerebral compartment of the head. Near-infrared light passes through the overlying scalp and skull tissue layers in order to interrogate the cerebral tissues underneath, and significant optical absorption and scattering can occur in these layers (Franceschini et al., 1998, Kirilina et al., 2012, Erdoğan et al., 2014).

It is important to understand and account for such potential confounds in order to reach reliable conclusions (Minati et al., 2011, Scholkmann et al., 2014b, Tachtsidis and Scholkmann, 2016). Numerous approaches have been proposed, ranging from purely statistical signal processing to biophysical modelling at various levels of detail.

Statistical models rest on the identification of shared variational relationships between different contributory elements in the measured signals. Importantly, systemic factors such as blood pressure and heart rate, along with contaminant estimators such as fNIRS recordings with short channel separations, may be included as additional regressors (Saager et al., 2011, Gagnon et al., 2012, Goodwin et al., 2014, Brigadoi and Cooper, 2015, Yücel et al., 2015).

In contrast, biophysical modelling approaches constrain system behaviour based on knowledge of the underlying physiology. Cerebral haemodynamics are affected by both active regulation and the passive biomechanics of the blood vessels and surrounding tissue, in turn constrained by the rigid enclosure of the skull (Zhang, 2002, Hu et al., 2006, Tzeng and Ainslie, 2013, Ainslie, 2014). Interactions between these elements and the response to neuronal activation are complex (e.g. (Maggio et al., 2014)) and have been modelled in numerous ways.

The system is usually considered as one or more conductive compartments that offer some resistance to flow and have some capacity to distend. A convenient analogy is to an electrical circuit, with blood flow corresponding to electrical current through resistors and volume to charge stored on capacitors. The Balloon (Buxton et al., 1998, Friston et al., 2000, Buxton et al., 2004) and Windkessel (Mandeville et al., 1999, Olufsen et al., 2002, Boas et al., 2003) models are archetypes of this form. Resistances and capacitances are not fixed and may have functional dependencies on flow, volume and other stimuli. An important foundation for treatments of the latter is the Ursino-Lodi family of models (Ursino and Lodi, 1997, Ursino and Lodi, 1998, Ursino et al., 2000), which are based on similar principles to the Balloon and Windkessel models but include influences from systemic factors such as blood pressure and blood ${\mathrm{CO}}_{2}$ concentration, as well as the production and reabsorption of cerebrospinal fluid. These models originate in the study of autoregulation and intracranial pressure rather than neuroimaging.

Modelled haemodynamics relate to fNIRS data via the quantities of marker species, particularly oxyhaemoglobin (${\mathrm{HbO}}_{2}$) and deoxyhaemoglobin (HHb), present in the imaged volume. The amounts of each change with blood flow in and out of the tissue and also with oxygen diffusion and consumption. Typically fNIRS-oriented models treat the imaged tissue as effectively homogenous, simply estimating the NIRS measurements from relative blood volume, but there have been a number of attempts to give a more detailed characterisation of the relationships between blood flow, tissue oxygenation and the optical signals (Fantini, 2002, Fantini, 2013, Fantini, 2014, Diamond et al., 2006, Diamond et al., 2009).

In this paper we use a modified version of the BrainSignals biophysical model (Banaji et al., 2008, Caldwell et al., 2015) to investigate confounding by systemic and extracerebral factors, with particular reference to the issue of misleading ‘false positive’ results, which have the appearance of activation when in fact none occurred, and ‘false negative’ results, which do not show evidence of activation even though it was actually present (Tachtsidis and Scholkmann, 2016). The existing model, a simplified descendant of the earlier BrainCirc (Banaji et al., 2005), addresses the cerebral compartment only. It incorporates both a haemodynamic component that models autoregulation and ${\mathrm{CO}}_{2}$ reactivity (drawing on (Ursino and Lodi, 1998)) and a model of a portion of the mitochondrial metabolism (drawing on (Korzeniewski and Zoladz, 2001)) to model oxygen consumption. Here we extend this with an additional compartment to model scalp haemodynamics.

The purpose of the joint model is to provide a tool by which the potential contributions to measured fNIRS signals can be understood and to assist the interpretation of experimental data that may be subject to confounding. This is in contrast to more ‘model-free’ denoising approaches, in which the systemic factors are directly regressed out of the measurements. While these approaches can be very successful (Saager and Berger, 2005, Tachtsidis et al., 2010b, Gagnon et al., 2014b), the implicit assumption that confounds map linearly to fNIRS artefacts may fail to capture more complex or interacting effects. Moreover, if the systemic changes are correlated to the cerebral activation there is a risk that some of the functional brain activity may be regressed out along with systemic contributions. A more explicit modelling approach allows the inclusion and exploration of key interactions governing system behaviour from known physiology. As the relationship between fNIRS measurements and systemic physiological parameters is often non-linear and non-stationary, this approach allows a better description of this complexity. In addition to providing a tool of data integration and denoising, this approach provides a test base platform for computational simulation investigations of various physiological scenarios such as the ones presented in this paper.

## 2 Methods

### 2.1 Modelling

**Fig. 1:**
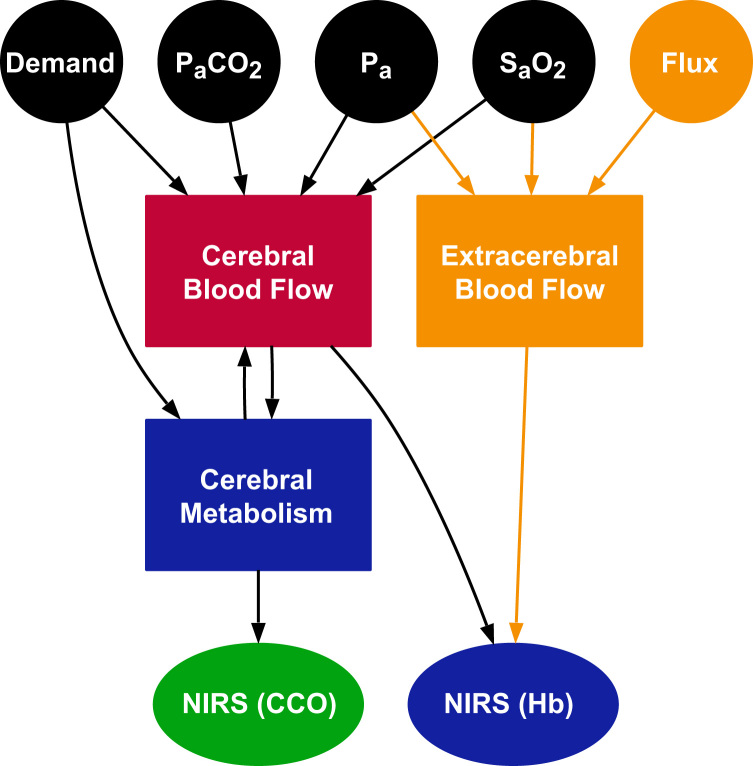
Main structure of the BSX model. Model inputs are represented as circles, the main dynamic compartments as rectangles and outputs as ellipses. Two distinct NIRS outputs are simulated: haemoglobin-based measurements (labelled Hb), estimated from the blood flow compartments, and measurements of the cytochrome *c* oxidase redox state (labelled CCO), estimated from the metabolic model. Elements shown in orange are new to BSX, while those in blue are modified from the previously published model B1M2 in Caldwell et al. (2015). The remaining elements are adopted unchanged. (A more detailed diagram showing the relationships between all variables and parameters in the model can be found in Supplementary Fig. 1.).

The model used here, termed BSX (from BrainSignals eXtended), derives from earlier models described in Caldwell et al. (2015) and Banaji et al. (2008). The overall structure shared by all these models is depicted in Fig. 1. There are two main interacting functional compartments: a haemodynamic compartment representing blood flow and oxygen delivery to the brain tissue, and a metabolic compartment, representing oxygen consumption in the neuronal mitochondria. There are important feedback relationships between the two compartments, since metabolism depends on the supply of $O_{2}$, while $O_{2}$ concentration and metabolic demand are among the modulators of blood flow. The state variables in the two compartments are used to predict NIRS measurements of haemoglobin and cytochrome *c* oxidase (CCO).

In the haemodynamic compartment, blood flow is driven by, and regulated in response to three systemic inputs—mean arterial pressure (P_a_), arterial partial pressure of carbon dioxide ($P_{a}{\mathrm{CO}}_{2}$) and arterial oxygen saturation ($S_{a}O_{2}$)—together with an explicit control parameter (*u*) representing relative metabolic demand. The latter may be increased to represent functional activation. The regulatory effects of the inputs follow the normal physiological responses: cerebral autoregulation to maintain blood flow as pressure varies, vasoconstriction and vasodilation in response to ${\mathrm{CO}}_{2}$ and $O_{2}$ changes. The blood flow modelling is dynamic, but does not attempt to capture fast (intra-beat) behaviour in any detail—this is reflected in the use of mean pressure as an input rather than the full pressure waveform. The input signals are not independently modelled—if not specified they remain at constant default values—so the model does not spontaneously produce behaviour such as slow pressure oscillations.

In the metabolic compartment, activity centres on a sequence of three redox reactions at the end of the mitochondrial electron transport chain, which may be limited by the oxygen supply. Importantly, the demand parameter *u* also affects these reactions via an influence on the electrochemical gradient for protons across the inner mitochondrial membrane.

**Fig. 2:**
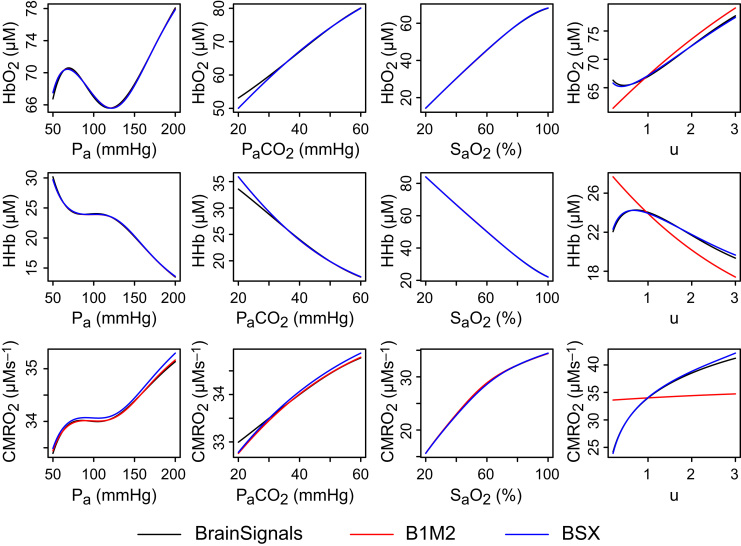
Steady state behaviour of the models under different inputs. Steady state outputs of oxygenated haemoglobin (${\mathrm{HbO}}_{2}$, top row), deoxygenated haemoglobin (HHb, middle) and cerebral metabolic rate of oxygen (CMRO_2_, bottom) for varying values of four model inputs: mean arterial pressure (*P_a_*, left column), arterial partial pressure of carbon dioxide ($P_{a}{\mathrm{CO}}_{2}$, centre left), arterial oxygen saturation ($S_{a}O_{2}$, centre right) and relative metabolic demand (*u*, right). Results from previously published model B1M2 correspond well with those of BrainSignals for the first three inputs, but the responses to changes in demand diverge significantly. To better capture these responses, which are important for modelling functional activation, the metabolic submodel in BSX has been modified as described in Eqs. (1), (2), (3) and the accompanying text.

The earlier publications described a number of variants within this overall structure, with the different elements represented in somewhat greater or lesser detail. BSX is largely based on the variant denoted B1M2 in Caldwell et al. (2015). In that variant, some of the biomechanical terms underlying the regulation of blood flow were substituted with a fitted linear model. This substitution is retained in BSX because it correctly reproduces the required behaviour in a tractable form (Fig. 2). However, the other simplification made in that version, to the metabolic submodel, did not produce the correct responses to variations in the demand parameter *u* (Fig. 2, right hand column). Since this is an important factor in the coupling between neuronal activity and blood flow, that modification was revised for BSX to produce the desired behaviour.

The core reactions of the metabolic submodel are those by which electrons are transferred (1) from a reducing substrate to the ${\mathrm{Cu}}_{A}$ centre of CCO; (2) from ${\mathrm{Cu}}_{A}$ to the $a_{3}$ centre; and finally (3) to $O_{2}$. Each of these reactions has an associated rate (denoted *f*_1_, *f*_2_ and *f*_3_, respectively) and these rates jointly determine the overall rate of oxygen consumption and energy production. Each rate depends on the availability of the electron donor and acceptor species for the reaction. Since the reactions entail the transfer of protons out of the mitochondrial matrix, the rates are also modulated by the proton motive force, Δp. For the BSX metabolic submodel, we define the rates using the following linear relations:

(1) $$f_{1}=\lambda_{f_{1}}+\lambda_{f_{1},p}\Delta p+\lambda_{f_{1},a}\log[{\mathrm{Cu}}_{A,\mathrm{ox}}]$$

(2) $$f_{2}=\lambda_{f_{2}}+\lambda_{f_{2},p}\Delta p+\lambda_{f_{2},a}\log[{\mathrm{Cu}}_{A,\mathrm{red}}]+\lambda_{f_{1},b}\log[a_{3,\mathrm{ox}}]$$

(3) $$f_{3}=\lambda_{f_{3}}+\lambda_{f_{3},p}\Delta p+\lambda_{f_{3},b}\log[a_{3,\mathrm{red}}]+\lambda_{f_{3},O}\log[O_{2}]$$

The coefficients $\lambda_{\left(\cdot \right)}$ represent the level of dependence on each variable, with the intercept $\lambda_{f_{\left(\cdot \right)}}$ representing the level independent of those influences. Note that the other variables have a non-zero contribution at baseline, so the intercept is not itself the baseline rate. All three rates include an indirect dependence on *u* via Δp. We have omitted a dependence on substrate supply on the assumption that it is not limiting; this should be reasonable for functional experiments with healthy volunteers. Coefficients were fitted as in Caldwell et al. (2015), but using expanded simulation data that included more extensive sampling of variations in *u*; for consistency, the unmodified parts of the model were also refitted from this data.

As shown in Fig. 2, the new model closely approximates BrainSignals behaviour for changes in *u*, as well as retaining the earlier version's simulation of responses to the other model inputs.

Scalp blood flow is known to vary with a number of factors, including cardiac output, ambient temperature, blood ${\mathrm{CO}}_{2}$ concentration and, most strongly, sympathetic nervous system (SNS) activity (Low et al., 1983, Khan et al., 1991, Drummond, 1991, Drummond, 1996, Drummond, 1997, Kashima et al., 2012). A degree of blood pressure autoregulation may occur, but much more weakly than in the cerebral vasculature (Wilson et al., 2005). Flow is not directly related to neural activity, although there may be indirect effects via the SNS, for example if the functional task is challenging or stressful (Kenney and Johnson, 1992, Hirasawa et al., 2015). A detailed model of scalp behaviour would need to account for many or all of these factors. However, the underlying processes are poorly understood and we do not in general have a sound basis for formulating or parameterising such a model. Moreover, doing so would certainly increase the data requirements of the model and place a greater burden on fNIRS experimenters.

In practice, the purpose of our modelling is not to predict scalp blood flow from first principles. Rather, we wish to estimate the contribution of that flow, regardless of its underlying causes, to the fNIRS signals. To do so we make a number of simplifying assumptions.

The scalp is modelled as a separate tissue compartment in parallel to the brain. Blood flow through this compartment is assumed to be dependent on a subset of the same systemic variables as the cerebral compartment, but conditionally independent of it—that is, behaviour of the cerebral compartment does not influence behaviour of the scalp compartment, and vice versa.

The relationships between pressure, flow and volume take a Windkessel-like form. Flow is assumed to be driven by pressure, *P*, across a variable conductance, *G*, with a relationship analogous to Ohm's Law:

(4) F=PG

The conductance, *G*, is assumed to be determined by blood vessel geometry, with the bulk of the resistance—and all its variability—exerted in the arterial/arteriolar vessels. This resistance is expressed in terms of a characteristic vessel radius, *r*, whose effect goes by Poiseuille's Law:

(5) $$G\propto r^{4}$$

We assume that the overall volume of arterial blood in the tissue, $V_{a}$, scales according to the vessel cross-sectional area:

(6) $$V_{a}\propto r^{2}$$

The venous vessels, conversely, are assumed to contribute a relatively small fixed resistance, but function primarily as a volume store via the portion of the blood pressure, $P_{v}$, acting over a constant venous compliance $C_{v}$:

(7) $$V_{v}=P_{v}C_{v}$$

This venous pressure, and consequently the volume of venous blood, varies over time with the applied systemic arterial pressure and the overall conductance.

(8) $$\frac{dP_{v}}{dt}=\frac{G(P-P_{v})-P_{v}G_{v}}{C_{v}}$$

We assume that the scalp arterial pressure is the same as the systemic arterial pressure *P*_*a*_ and that the post-venous pressure is negligible. Venous compliance is based on an estimate from Olsen and Länne (1998). A control parameter, ${\mathrm{Vol}}_{c,\mathrm{frac}}$, specifies how much of the baseline venous volume is due to compliance. A second control parameter, $R_{\mathrm{frac},v}$, is used to specify the fraction of overall vascular resistance we expect to reside in the venous compartment at baseline. By default both parameters are set to 0.1 (10%). In practice, the dynamic behaviour of the model is relatively insensitive to these values provided that the general scale assumptions are preserved—i.e., $P_{v}\ll P_{a}$.

In order to calculate *G*, we require an estimate of the blood flow *F*. We adopt two distinct approaches for *F*, depending on the instrumental data available.

In the case where we do not have a direct measure of flow, we make the assumption that the flow is directly dependent on the pressure via the linear relation

(9) $$F_{x}=\lambda_{F_{x}}+\lambda_{F_{x,p}}P_{a}$$

We refer to this as the ‘pressure-based’ scalp model, and add the subscript *x* to distinguish variables and parameters specific to it. This is clearly a very substantial simplification relative to the possible driving factors discussed above and we would expect to produce only a partial approximation of the true flow. Nevertheless it captures at least some of the scalp susceptibility to systemic drivers without adding to the data burden, since *P*_*a*_ is already required by the scalp model. Additional systemic variables could be added to this model if sufficient data were available for parameterisation. In this case, the parameters $\lambda_{F_{x}}$ and $\lambda_{F_{x,p}}$ were determined by fitting the linear model to experimental data published in Gagnon et al. (2014a).

The second approach applies when scalp flow has been measured by laser Doppler (LD) as part of the experimental protocol. The LD measurement, often referred to as ‘flux’, does not provide an absolute measure of flow, so for the purposes of the model it is normalised to an initial baseline level determined from the data. We assume the resulting quantity represents flow changes, such that an estimate of flow can be obtained by multiplication with a ‘normal’ value:

(10) $$F_{y}={\mathrm{Flux}}_{y}\times F_{y,n}$$

We refer to this as the ‘flux-based’ scalp model, and use the subscript *y* to distinguish its variables and parameters. The normalised value, *Flux*_*y*_, is added as a new model input, while the normal value, $F_{y,n}$, is a parameter. For the purposes of simulations in this paper, we have chosen a value that matches the baseline value arising from the fit of the pressure-based model. This simplifies comparison between the models, but may be a source of errors in the predictions from individual data.

No attempt is made to model metabolism in the scalp compartment and oxygen consumption is assumed to be fixed. Arterial and venous volumes are considered homogenous, with no spatial variation or transit time. Oxygen saturation throughout the arterial volume is assumed equal to the systemic arterial saturation, while venous saturation is equal to the baseline venous saturation of the cerebral model. Both arterial and venous volumes are normalised to a reference volume such that at baseline conditions the total volume is 1 and the venous-arterial ratio is at a set level, by default 3:1. Variations in both volumes are used to estimate the NIRS haemoglobin outputs HHb and ${\mathrm{HbO}}_{2}$ by scaling for haemotocrit and blood vessel density. These parameters are set to be consistent with those in the cerebral model.

To represent the effect of extracerebral confounding for the NIRS signals, we add new merged ${\mathrm{HbO}}_{2}$ and HHb outputs, which are simply a weighted sum of the corresponding outputs from the cerebral and scalp models. These are primarily for use during model optimisation for ‘false negative’ and ‘false positive’ scenarios (Section 3.2) and do not affect the model behaviour. Separate NIRS signals for each compartment are also generated, and these are used in analysis and attribution of experimental data in 3.3, 3.4.

All models were implemented using the Brain/Circulation Model Developer (BCMD) environment (https://github.com/bcmd/BCMD). The complete model definition in textual form, with all equations and parameters, is provided in Supplementary Data 1, and its structure depicted graphically in Supplementary Fig. 1. Model implementation files, data files and the scripts used for analysis are freely available from http://dx.doi.org/10.5281/zenodo.56569.

### 2.2 fNIRS data

The model was applied to example functional NIRS data drawn from earlier studies by Kolyva et al. (2012) and Scholkmann et al. (2013a). Here we summarise the salient experimental features; for more detailed descriptions of the methods, see the cited papers.

In the 2012 study, 11 healthy adult subjects attempted to solve a sequence of anagrams presented on computer screen, without verbalising their answers. The active period of the task was 6min, with 2min baseline recording before and after. Arterial blood pressure and heart rate were measured with a Portapres monitor (Finapres Medical Systems, Netherlands), and scalp blood flow with a laser Doppler probe (Moor Instruments, UK) placed on the forehead. NIRS data were recorded using a custom hybrid optical spectrometer including both frequency domain (FD) and broadband components (Tachtsidis et al., 2010a). The broadband channels included source–detector separations of 2.0, 2.5, 3.0 and 3.5 cm, while the FD used only 3.0 and 3.5 cm. Optodes were placed on the right side of the forehead, over prefrontal cortex, region Fp2 in the 10–20 placement system. Relative tissue concentrations of oxygenated and deoxygenated haemoglobin ($\Delta {\mathrm{HbO}}_{2}$ and ΔHHb) were fitted from the NIRS measurements with the UCLn algorithm (Matcher et al., 1995). All data were resampled to a uniform sampling interval of 3 s.

The 2013 data were obtained from 24 healthy adult subjects performing (on separate days) 3 different speech tasks and one mental arithmetic task. Tasks were performed for two 5 min periods, with an initial baseline recording period of 8min, a 5min recovery interval between the two task periods, and final recovery period of 20min. Heart rate was recorded with a Medilog AR12 Plus monitor (Schiller AG, Switzerland) and $P_{\mathrm{et}}{\mathrm{CO}}_{2}$ with a Nellcor N1000 gas analyser (Covidien Medtronic, MN, USA). NIRS data were recorded with an OxiplexTS FD spectrometer (ISS, IL, USA). Optodes were placed on both right and left sides of the forehead, in 10–20 placement regions Fp2–4 and Fp1–3 respectively, with source–detector separations of 2.0, 2.5, 3.0 and 4.0 cm. Haemoglobin signals were calculated in the OxiplexTS software using the frequency domain multi-distance method (Hueber et al., 2001). The multi-distance geometry was also used to compensate for confounding from superficial layers (Franceschini et al., 1998, Choi et al., 2004) and to reduce movement artefacts (Scholkmann et al., 2014c).

### 2.3 Signal processing and data analysis

Model simulations based on synthetic input data were run with an output sampling rate of 1 Hz and no filtering was performed on the generated signals. Systemic and NIRS data from Scholkmann et al. (2013a) were supplied in group averaged form at a sampling interval of 1 min. This was upsampled to 1 Hz by linear interpolation. Model outputs were again generated at 1 Hz and not post-filtered. Systemic and NIRS data from Kolyva et al. (2012) were provided at a sampling interval of 3 s. These were not resampled and model outputs were generated at the same 3 s interval. In the original 2012 analysis these data were smoothed with a 1 min sliding window average, which also served to reduce a prominent Mayer wave component at ∼0.1Hz (Julien, 2006). Both for consistency with the original data and because this component could obscure underlying functional responses in some records, the same 1 min moving average smoothing was applied to model outputs for these data sets. Of the 11 subjects recorded in the original study, 3 were excluded because systemic data were not recorded. In contrast to the original analysis, subjects were not excluded based on the absence of an identifiable functional activation response.

Laser Doppler flux measurements were normalised to a baseline calculated as the median value during the initial 2 min recording period prior to the start of the anagram task. Dynamic Time Warping was performed using the R dtw package (Giorgino, 2009). Model optimisation for false positives and false negatives was done using the Galileo genetic algorithm in OpenOpt (http://openopt.org) via the batch facilities of BCMD. A model extension to generate the step inputs was implemented for this purpose; this extension and the accompanying optimisation scripts are included with the model distribution (doi:10.5281/zenodo.56569). Attribution of NIRS signals to the brain and scalp components was performed using the optim function in R to minimise the Euclidean error, imposing non-negativity constraints with the L-BFGS-B method.

All new data analysis, signal processing and plotting was performed in R (Team, 2013). Experimental data from Scholkmann et al. (2013a) and Kolyva et al. (2012) had previously been processed using equivalent methods in Matlab (Mathworks, Natick, MA, USA). Figures were assembled in Illustrator CC 2015 (Adobe, San Jose, CA, USA) and the text was prepared using LATEX (http://tug.org).

## 3 Results

### 3.1 Simulations with synthetic data

BSX (Fig. 1) defines five input variables to represent systemic factors. These are listed in Table 1, together with their baseline values and the range of values admitted in our simulations as ‘normal’.

**Fig. 3:**
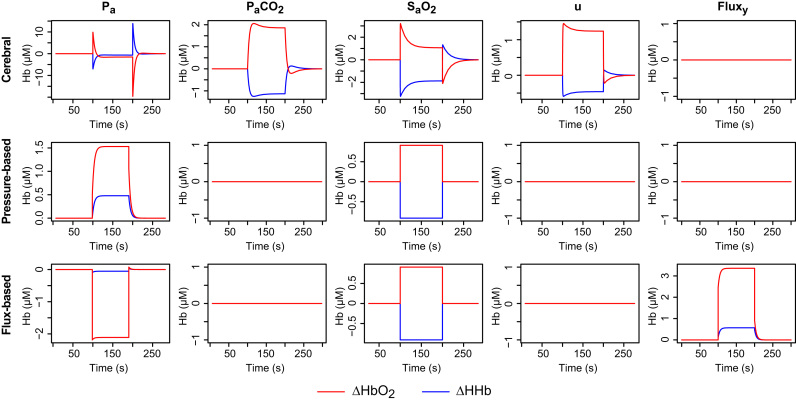
Simulated haemodynamic responses to step changes in a single input variable (by column, left to right): mean arterial pressure (*P_a_*); arterial partial pressure of ${\mathrm{CO}}_{2}$ ($P_{a}{\mathrm{CO}}_{2}$); arterial oxygen saturation ($S_{a}O_{2}$); metabolic demand (*u*); normalised scalp blood flux (*Flux_y_*). In each case, the step is between the baseline and upper bound values given in Table 1. Modelled responses are shown separately for the cerebral compartment (top row), the pressure-based extracerebral model (middle row) and the flux-based extracerebral model (bottom row).

To illustrate the basic responses of the different model compartments, the model was first driven with synthetic inputs consisting of isolated step changes in each of the systemic variables. Such changes are non-physiological but provide a useful reductive test of model behaviour. In each case, the input variable was stepped from the baseline to the upper level listed in Table 1, and then back to the baseline. Plots of the resulting changes in ${\mathrm{HbO}}_{2}$ and HHb are shown in Fig. 3.

The responses to the stimuli reflect the different features of the models. In the cerebral model, a change in blood pressure is quickly compensated by autoregulation, which decreases conductance to reduce flow. This results in a reduction in arterial volume and hence a relative decrease in ${\mathrm{HbO}}_{2}$. The increases in *u* and $P_{a}{\mathrm{CO}}_{2}$ both drive an increase in conductance, with some delay, which then reduces again more slowly as the increased flow leads to a rise in tissue oxygen. A saturation increase appears in the simulated NIRS immediately, since the arterial vessels are assumed all to be at the same saturation, but again the conductance is slowly reduced in response to increasing tissue oxygen until supply and demand are in balance.

Since the scalp models do not include any regulation, their time courses are simpler. Saturation changes again appear immediately, producing a simple step response. Flux and pressure changes are buffered by venous compliance, converging exponentially to a new steady state value. It should be noted that, because the flux model assumes that flow is driven by pressure, an increase in pressure with no accompanying change in flux is interpreted as a reduction in conductance. As seen in the bottom left plot of Fig. 3, this manifests as a drop in the haemoglobin signals. However, this behaviour is primarily an artefact of the isolated changes in these simulations, which we would not expect to see in practice.

**Fig. 4:**
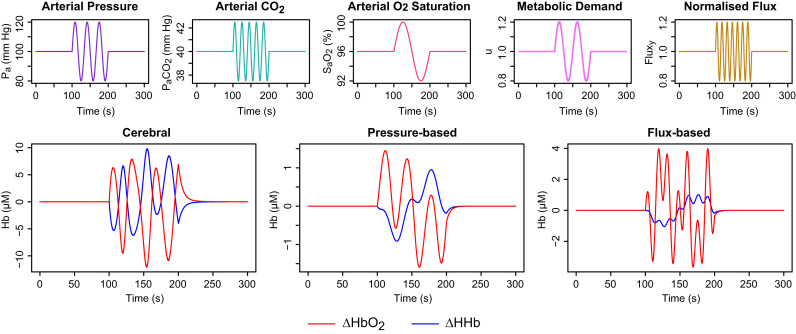
Simulated haemodynamic responses when all model inputs are varied simultaneously. The time course of each input is shown in the upper row, while the modelled haemoglobin signals for the cerebral and scalp compartments are shown in the lower row. The modelled compartments exhibit markedly different haemodynamic responses due to the differing input dependencies and interactions. We would expect the differences to be less obvious in real experimental data but some degree of separability is plausible.

In practice, systemic factors are unlikely to vary in isolation, and a more interesting test scenario is that presented in Fig. 4. All five inputs were varied simultaneously to illustrate the different responses of the model compartments. The input waveforms are depicted in the top row of the figure. The oscillatory segment of each input has a different frequency, so the inputs are linearly independent.

Because each blood flow model is driven differently by the inputs, the outputs generated (bottom row of Fig. 4) are also linearly independent. In such a case, the signals should be separable and we ought to be able to estimate their relative contributions in measured data. In practice, the inputs are unlikely to be independent and the data will also be noisy and incomplete, so true separability is unlikely. Nevertheless, we may be able to produce an approximate attribution in some cases.

### 3.2 Systemic factors can mask or mimic functional activation

Given that systemic factors can have opposing effects on cerebral haemodynamics and oxygenation, it has been suggested that experimental data may be susceptible to artefacts open to misinterpretation (Tachtsidis et al., 2009, Scholkmann et al., 2013a, Tachtsidis and Scholkmann, 2016). To investigate the plausibility of this concern, we applied the model to see whether and how such misleading outputs could be generated.

**Fig. 5:**
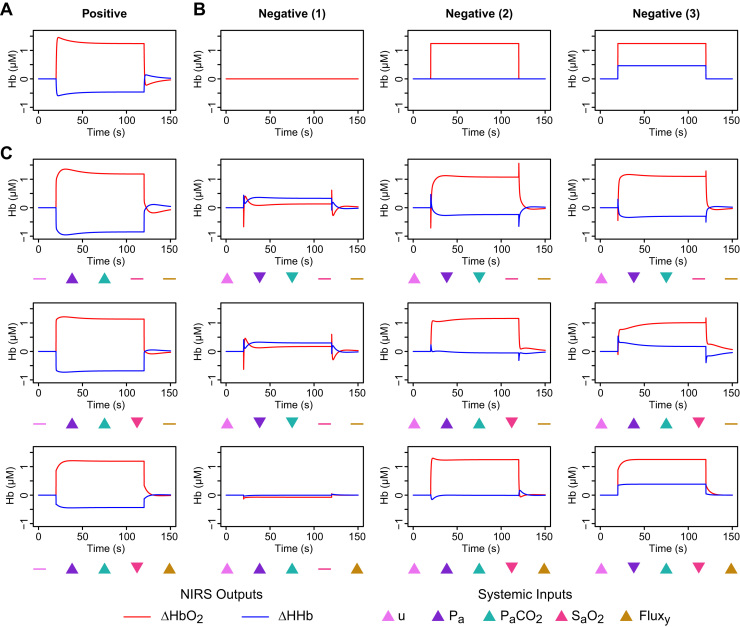
Simulated false positives and false negatives arising from systemic and extracerebral contamination. **A** Haemodynamic response characteristic of functional activation. **B** Example responses that would not be identified as functional activation. **C** Simulated responses optimised to resemble the corresponding plots above in panel A (with no activation) and B (with the same level of activation as in panel A). In the first row of panel C, only arterial pressure and ${\mathrm{CO}}_{2}$ were allowed to vary. In the middle row, a drop in arterial $O_{2}$ saturation was also permitted. In the bottom row, a contribution from the scalp compartment was also included and the superficial blood flux allowed to vary. Markers below each plot indicate the direction of change of the systemic variables giving rise to the observed signals. (More detailed plots are provided in Supplementary Data 2. Individual values for all the inputs in each case are provided in Supplementary Data 1.).

Functional activation is classically associated with a haemodynamic response like the one depicted in Fig. 5A: a rise in ${\mathrm{HbO}}_{2}$ accompanied by a decrease in HHb (Scholkmann et al., 2014b). This plot is indeed the response produced by the model in response to an increase in the demand *u* with no accompanying change the systemic inputs. Without such a response, conversely, no functional activation will be detected. For optimisation purposes we specify three different scenarios that would typically be classified as a lack of activation, shown in Fig. 5B. In the first and simplest case, there is no haemodynamic change at all, while in the second and third there is an increase in ${\mathrm{HbO}}_{2}$ but HHb either does not change (scenario 2) or also increases (scenario 3). Historically, some investigators have chosen to use only the ${\mathrm{HbO}}_{2}$ signal (for recent examples see Balconi et al., 2015, Brucker et al., 2015), and might thus interpret the latter cases as evidence of activation. However, the HHb response is generally acknowledged to have greater specificity and the use of both signals in conjunction is preferred (Tachtsidis and Scholkmann, 2016).

Numerical optimisation was performed to find combinations of input changes that could generate *false positive* (FP) outputs—resembling Fig. 5A despite the absence of genuine functional activation—and *false negative* (FN) outputs—resembling the scenarios of Fig. 5B despite an actual increase in metabolic demand. Some example results are shown in Fig. 5C. Note that these optimisations are not convex and the genetic algorithm is non-deterministic, so the results are neither unique nor necessarily optimal. Similar responses could be obtained in other ways.

It can be seen that both FP and FN responses can be provoked by changes in $P_{a}{\mathrm{CO}}_{2}$ and arterial pressure, although optimisation has only managed to mimic FN type 1 and the approximation is imperfect. If a scalp contribution is also included (Fig. 5C, bottom row) then all three FN scenarios are reproduced persuasively.

Note that the idealised nature of the simulations—instantaneous, synchronous step changes, with no filtering, jitter or noise—in some cases leads to transients or other temporal features that might seem, at least in principle, to allow the different cases to be distinguished. However, in practice instantaneous changes will not occur, and such markers are unlikely to be identifiable against the background of physiological and instrumental variation.

**Fig. 6:**
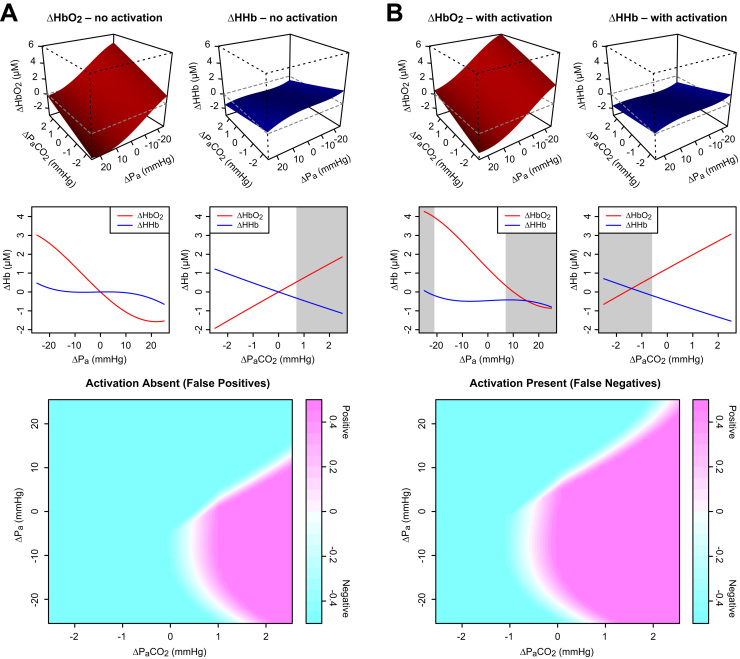
Variation of haemodynamic responses with changes in $P_{a}{\mathrm{CO}}_{2}$ (from baseline 40 mmHg) and blood pressure (baseline 100 mmHg). In the upper 3D graphs, responses are shown for simultaneous changes in both inputs. (Note the axis for $P_{a}$ in these plots increases from right to left.) The 2D graphs in the middle row show effects of changing $P_{a}$ and $P_{a}{\mathrm{CO}}_{2}$ alone. Shaded regions indicate where a false identification results when using a simple distance-based classifier. At the bottom, results from the same classifier are shown for both factors varying jointly over their ranges, with colour indicating the strength of the classification. **A** Systemic factors were varied with no change in metabolic demand. False positive responses (magenta in the bottom plot) occurred in the region of reduced pressure and increased ${\mathrm{CO}}_{2}$. They could be produced by ${\mathrm{CO}}_{2}$ changes alone, but not by pressure. **B** Metabolic demand was increased at the same time as the systemic changes, shifting the ${\mathrm{HbO}}_{2}$ response upwards and the HHb downwards. False negatives (cyan in the bottom plot) occurred across a wide range of combinations, but especially with reduced ${\mathrm{CO}}_{2}$ and increased pressure. They could also arise from changes in either factor alone.

To investigate further which changes in blood pressure and $P_{a}{\mathrm{CO}}_{2}$ might lead to misleading responses, additional step change simulations were performed across the ‘normal’ ranges of both inputs, both with and without a corresponding change in *u*. The changes were again simultaneous, but the transient time course details were ignored and only the final steady-state values of $\Delta {\mathrm{HbO}}_{2}$ and ΔHHb after the step taken into account. Outputs were classified according to their proximity (lowest total absolute error) to the target signals of Fig. 5A–B. The results are shown in Fig. 6.

Increasing arterial pressure leads to a reduction in both ${\mathrm{HbO}}_{2}$ and HHb (we assume the pressure remains within the range of functioning autoregulation), while increasing $P_{a}{\mathrm{CO}}_{2}$ increases ${\mathrm{HbO}}_{2}$ and decreases HHb. The latter profile corresponds to what we look for as characteristic of functional activation, so it appears that hypercapnia alone may be sufficient to generate FP results, while pressure changes alone cannot (Fig. 6A).

In the presence of true functional activation (Fig. 6B), the effects of $P_{a}{\mathrm{CO}}_{2}$ and arterial pressure are similar but the response surfaces are shifted. Since a false negative may arise in more than one way—by suppressing the functional ${\mathrm{HbO}}_{2}$ increase, or the HHb decrease, or both—we conclude that both $P_{a}{\mathrm{CO}}_{2}$ and pressure could in principle lead to FNs.

### 3.3 Systemic confounding in experimental data

As a practical example of systemic confounding in a functional setting, we used our model to re-examine data from Scholkmann et al. (2013a). In that study, end-tidal ${\mathrm{CO}}_{2}$ ($P_{\mathrm{et}}{\mathrm{CO}}_{2}$, a widely-used non-invasive surrogate for the arterial ${\mathrm{CO}}_{2}$) was measured alongside fNIRS during a number of spoken language tasks. A number of investigators had previously observed uncharacteristic fNIRS and BOLD-fMRI responses during speech tasks and it was hypothesised that ${\mathrm{CO}}_{2}$ confounding might underlie those results.

For the data examined here, both the fNIRS and ${\mathrm{CO}}_{2}$ signals were averaged over many subjects, and NIRS also over left and right brain hemispheres, leading to significant smoothing of these time profiles compared to the sharper profile of the synthetic input. Nevertheless it is striking that the haemodynamic behaviour is not what we would expect for functional activation.

**Fig. 7:**
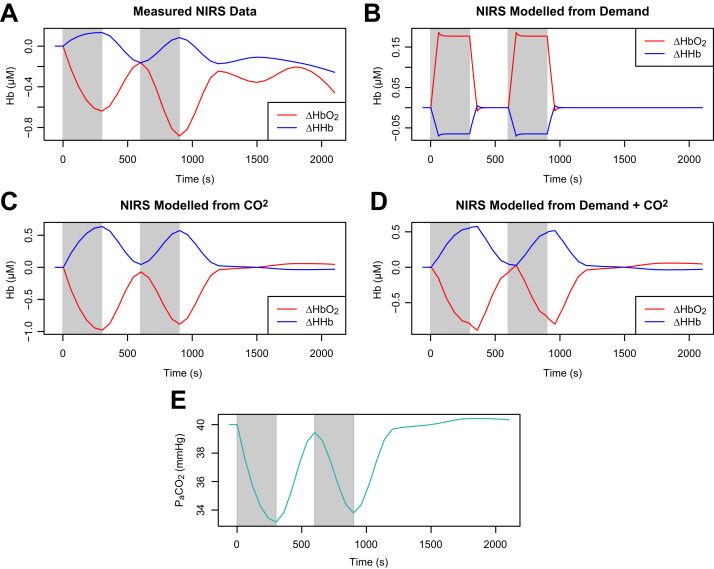
**A** Measured NIRS signals from a speech task do not exhibit expected functional activation haemodynamics. The response is very poorly modelled by increased metabolic demand (**B**), but much better modelled by ${\mathrm{CO}}_{2}$, either alone (**C**) or in combination with demand (**D**). **E** The measured ${\mathrm{CO}}_{2}$ data.

Measured ${\mathrm{HbO}}_{2}$ and HHb for one of the tasks (prose recitation) are shown Fig. 7A. If we attempt to model this behaviour with an increase in metabolic demand (Fig. 7B), the results are entirely unlike the data, with haemoglobin changes in the opposite direction. However, the data can be relatively well modelled using the measured ${\mathrm{CO}}_{2}$ (Fig. 7C). Adding increased demand as a factor alongside ${\mathrm{CO}}_{2}$ makes some difference, though it is relatively marginal (Fig. 7D).

It should be noted that the modelled variability closely resembles that of the ${\mathrm{CO}}_{2}$ data itself (Fig. 7E). The aggregation and timescale of the data relative to the ${\mathrm{CO}}_{2}$ response mean that temporal differences are relatively insignificant. In the absence of additional inputs there are also no interactions such as those between blood pressure and ${\mathrm{CO}}_{2}$ in the previous section. A similar profile could be obtained by attempting to fit the NIRS signals to the ${\mathrm{CO}}_{2}$ data by linear regression. However, this would only identify the correlations—it would still be necessary to explain them.

It is clear that the NIRS data in this case cannot reasonably be explained by functional activation. An interpretation which attempted to do so without taking into account the effects of ${\mathrm{CO}}_{2}$ would certainly be misleading. The evidence of the model reinforces the original paper's contention that recording ${\mathrm{CO}}_{2}$ levels in such experiments is advisable if incorrect inferences are to be avoided.

### 3.4 Extracerebral confounding in experimental data

To investigate extracerebral confounding with the model, we used data from a study published by Kolyva et al. (2012), in which (for 8 of the 11 subjects) the mean arterial pressure and LD flux were recorded in addition to broadband NIRS at multiple source–detector separations, during an anagram-solving task. As reported in the original paper, the subjects in this study exhibited heterogeneous responses, so we examined individual data sets rather than group averages.

The model was driven using the recorded systemic data for each individual, together with a defined increase in demand during the task period. We did not attempt to quantify a difference in demand between the alternating 4-letter and 7-letter anagram phases within the task. Since LD data were available, we used the flux-based version of the scalp model. Model outputs were compared with the NIRS measurements at the shortest and longest source–detector separations (2.0 and 3.5 cm respectively). It was expected that the longer distance data would include a greater contribution from cerebral tissue and less from the scalp (Saager and Berger, 2005, Brigadoi and Cooper, 2015).

Similarity between the modelled and measured signals was assessed by several measures. The Pearson correlation coefficient is a convenient indicator of similarity when signals are well aligned. In practice, the physiology can introduce variable delays that the model does not capture. To obtain a broader measure of the signal similarity, cross correlation was performed, noting the maximum correlation for each signal and the lag at which it occurred. As a further test, we applied the more flexible Dynamic Time Warp (DTW) method, in which signals are aligned using non-constant lags to minimise the distance between them (Giorgino, 2009). The similarity of signal ‘shape’ was assessed by the degree of warping required for this minimisation (computed as the mean distance between the two index vectors) as well as the final distance achieved. These metrics are non-negative by definition, with smaller values better; zero for both would indicate a perfect match.

It must be noted that there are differences of scale between the signals, both in the measurements at different distances, arising from the different tissue volumes interrogated, and in the cerebral and scalp compartment models, due to differences in the modelled behaviour and to uncertainties in some parameters. While correlations are insensitive to scale, this is not the case for DTW. In order to ensure that signals were compared on an equal footing, the modelled signals were rescaled into the same value range as the measurements before the DTW was performed.

**Fig. 8:**
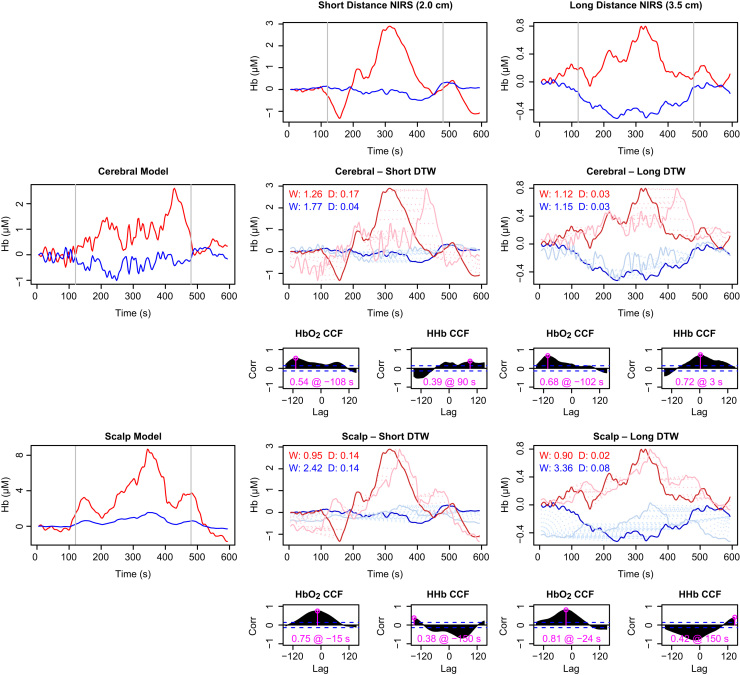
Comparison of NIRS data with model predictions for subject S8, showing a ‘normal’ response recognised as functional activation. Recorded haemoglobin signals at short and long source–detector separations are shown in the top row. Model predictions for the cerebral and scalp compartments are shown in the left column. Pairwise comparisons are shown in the corresponding rows and columns in the remainder of the figure. For each pair, the main plot depicts dynamic time warp (DTW) results for both ΔHbO_2_ and ΔHHb, while the two smaller plots below show the cross-correlation (CCF) between the real and modelled data for lags of up to 2.5 min. The maximum correlation, and the lag at which it occurs, is marked in pink on each CCF plot. In the DTW plots, the bold traces show the measured data and the fainter ones the (rescaled) model outputs. The dashed lines connecting the two show the warping between the two signal—longer lines thus indicate a poorer fit. The degree of warping (W) and final distance (D) are noted on the DTW plots in red for ${\mathrm{HbO}}_{2}$ and blue for HHb (for both metrics, smaller is better). Notably, the scalp model predicts ΔHbO_2_ better at both distances, but predicts ΔHHb very poorly. The modelling suggests this data set does indeed exhibit activation.

For subjects exhibiting the characteristic haemodynamics of functional activation, there is a broad agreement between the model predictions and the measured signals, although the variability in all measurements leads to many differences in detail. An example individual is shown in Fig. 8. As expected, the measurements made with shorter source–detector separation more closely resemble the scalp compartment, whereas at the longer distance there appears to be more contribution from the cerebral tissue.

Because the scalp model does not include active consumption it is unable to produce *opposing* changes in ${\mathrm{HbO}}_{2}$ and HHb without changes in saturation, which are not recorded here (and in any case unlikely). Thus, ΔHHb in the scalp model cannot fit the profile for functional activation and is anti-correlated with the recorded data for subjects like S8. Because the blood saturation is normally high (and is assumed to be so here, since we do not have explicit measurements), the HHb changes in the scalp compartment are smaller than those for ${\mathrm{HbO}}_{2}$. Nevertheless, the effect of extracerebral confounding would be to obscure the cerebral HHb drop.

**Fig. 9:**
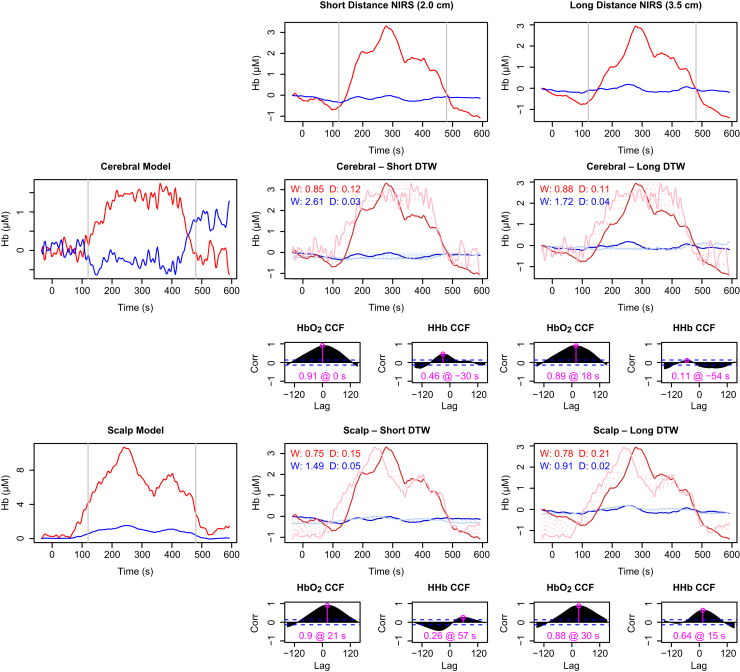
Comparison of NIRS data with model predictions for the subject S2, whose data did not meet the criteria the functional activation. Panel layout is the same as for Fig. 8. The ΔHbO_2_ signal is well-explained by both scalp and brain models. The ΔHHb response is marginally positive, so may be better explained by the scalp model, although the scale of this response in all cases is small relative to the signal variability and the correlations are poor. The modelling is not conclusive, but it does suggest that the negative classification in this case may be false.

Fig. 9 shows results from a subject classified as *not* exhibiting functional activation. In this case the $\Delta {\mathrm{HbO}}_{2}$ response is consistent with both the scalp and cerebral models. Measured HHb does not show substantial changes, but is somewhat better modelled by the scalp compartment. It is thus *possible* that the lack of an identified HHb response is an example of a false negative. In the absence of additional data we cannot conclude one way or the other, but the uncertainty is illustrative.

**Fig. 10:**
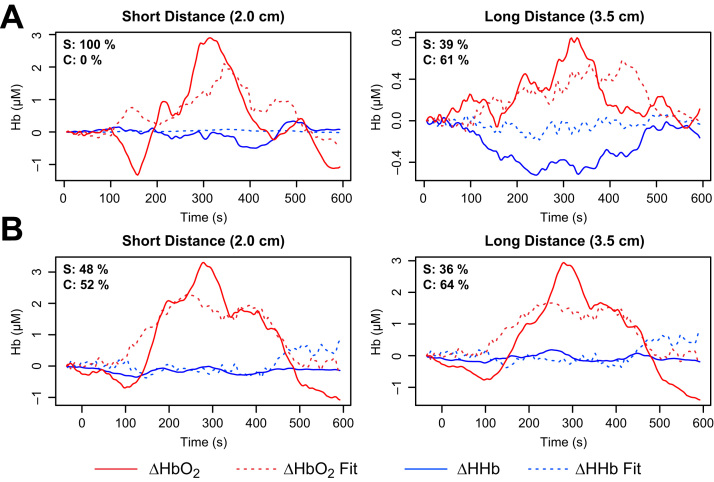
Estimating cerebral and scalp contributions to measured data. Measured signals (solid lines) were fitted as a sum of non-negative weighted contributions from the two modelled compartments, minimising total error over both ${\mathrm{HbO}}_{2}$ and HHb. Dashed lines show the fitted signals, and the values in the top left corner of each plot show the relative contributions of the scalp (S) and cerebral (C) compartments to the fits. **A** (Subject S8, as in Fig. 8.) At short distance, the best fit contains no cerebral contribution at all, while at the longer distance the scalp component is less than the cerebral. **B** (Subject S2, as in Fig. 9.) As would be expected, the short distance fit includes a larger scalp contribution than the long distance. It is notable that some (activated) cerebral contribution is included at both distances, despite the lack of a characteristic functional activation response in the HHb.

In general, NIRS measurements interrogate a volume including both scalp or cerebral components in unknown proportion. To attempt to assess the relative contributions in these recordings, the measurements were fitted as a non-negative weighted sum of the modelled signals, minimising the total error over both the ${\mathrm{HbO}}_{2}$ and HHb signals, with no lag or warping. We assumed both signals would be contaminated by scalp to the same degree. Examples of this attribution for the subjects of Fig. 8, Fig. 9 are shown in Fig. 10.

Because of uncertainties in scaling and timing of the modelled signals, as well as the simplistic cost function, these results must be treated with caution. It is unlikely, for example, that the short distance measurements in Fig. 10A truly contain only scalp changes. The estimate indicates only that whatever improvement might be achieved in the HHb signal by adding a contribution from the cerebral model would be outweighed by a simultaneous worsening of ${\mathrm{HbO}}_{2}$. Nevertheless, it is notable that in each case the fit identifies a lower scalp contribution at longer source–detector separation. In the context of the possibility that the S2 data shows a false negative, it is suggestive that the fit at both distances (Fig. 10B) includes a non-zero contribution from a cerebral model in which activation actually occurs. Again, we cannot infer that this is a genuine FN case but we also cannot rule it out.

## 4 Discussion

We have used a modified version of the BrainSignals physiological model, termed BSX, to investigate the susceptibility of functional neuroimaging to confounding by systemic factors and extracerebral tissues. While extracerebral influences are particularly relevant to fNIRS, systemic confounding is also of utmost concern for other haemodynamics-based modalities such as BOLD-fMRI.

The BSX model includes a representation of several factors that modulate cerebral blood flow, including systemic blood pressure and ${\mathrm{CO}}_{2}$ concentration. It is known that variations in such systemic factors can occur during functional experiments (Tachtsidis and Scholkmann, 2016). Moreover, in some cases there may be causal relationships between such variations and the performance of particular kinds of task, such as speech, which could give rise to *systematic* confounding.

We have used BSX to demonstrate that variations in systemic factors are able to induce haemodynamic responses that both mimic functional activation in its absence, to produce a false positive (FP) identification, and mask its occurrence when present, to produce a false negative (FN) result. These false outcomes occur over a range of possible values for the driving factors that remain well within the bounds of their normal physiological variability. Our simulated scenarios are idealised and limited by the simplifications inherent in the model, but arguably the additional behavioural complexity and sources of variation in real experimental data make false identifications even more difficult to exclude.

By definition, the extent to which FP and FN results occur in real experiments is not known. But as a concrete example we applied the model to group average data from Scholkmann et al. (2013a), a study in which the confounding influence of ${\mathrm{CO}}_{2}$ was specifically addressed. Our model results closely accord with the conclusions of that paper. The recorded haemodynamics appear to have been driven almost entirely by respiratory changes, to the extent that any neurovascular coupling that may also have occurred could not be identified.

For fNIRS experiments, it is plausible that blood flow in the scalp and superficial tissues may also contribute to FP and FN results. To allow investigation of this, BSX incorporates a rudimentary model of such extracerebral blood flow. Applying the model to individual data from Kolyva et al. (2012), we found that a variable but significant fraction of the observed haemodynamic behaviour could be attributed to the extracerebral compartment. Our results suggest that the HHb signal may be particularly susceptible to such contamination, because its response to activation is smaller and may be more easily obscured by parallel changes to superficial blood flow. However, the lack of active consumption in the scalp model may lead it to overestimate the impact on the ${\mathrm{HbO}}_{2}$ signal and underestimate that on HHb.

These results are only partial, because neither data set includes all of the important systemic variables. It is possible that blood pressure may also play a role in the speech results, although the respiratory impact of vocalisation provides a plausible explanation for the involvement of ${\mathrm{CO}}_{2}$. Similarly, we cannot rule out a role for ${\mathrm{CO}}_{2}$ in the anagram results. While it is not immediately obvious what that role might be, it is known that unvocalised language tasks can induce changes in blood oxygenation and ${\mathrm{CO}}_{2}$ (Scholkmann et al., 2014a). In either case, more complete systemic data would allow a better assessment to be made of the extent to which these factors play a role in the fNIRS signals.

The impact of blood pressure and ${\mathrm{CO}}_{2}$ on haemodynamics-based functional measurements is likely to vary substantially between different experimental procedures, but it is clear that they have the potential to exert a powerful influence. Both factors may be recorded relatively easily with modern instrumentation. Our model results support the argument that such recording is desirable to identify potential confounding. In the context of fNIRS, a similar case can be made for recording Laser Doppler flux measurements, since superficial blood flow effects are a recognised potential confounder.

Some aspects of the BSX model are deliberately simplistic and there is plenty of scope for future development. In particular, the scalp blood flow model neglects the influence of numerous factors that may impact on skin blood flow, notably the influence of the autonomic nervous system, an important factor affecting skin blood flow. A number of surrogates for autonomic activity, including heart rate, breathing rate and skin temperature, can be monitored, and inclusion of such factors in the model might allow for improved behavioural simulation in this compartment. The addition of an oxygen extraction function in the scalp may be beneficial to mitigate the risk of overestimating the effect on ${\mathrm{HbO}}_{2}$ relative to HHb. Both scalp and cerebral compartments currently make use of a simple homogenous measurement model. Much more detailed models of the relationship between optics, blood flow and oxygenation are available and might productively be incorporated into BSX.

While we have used the model to identify likely confounding and attribute behaviour among different causes, one potential use that has yet to be explored is that of explicit artefact-removal or denoising. Regression-based approaches to the removal of systemic or superficial confounds from fNIRS data have been demonstrated previously (Saager and Berger, 2005, Tachtsidis et al., 2010b, Gagnon et al., 2014b), but are limited in their ability to detect interacting and non-linear effects. Combining such methods with our model's ability to account for the ‘normal’ behaviour arising from systemic factors should allow researchers to focus more clearly on the experimentally-relevant functional components of the fNIRS data and thus better interrogate its neurophysiological significance.

In addition, we have so far only demonstrated this approach using data from studies with healthy adult volunteers. It is worth noting that the model's capacity to simulate physiological mechanisms such as cerebral autoregulation and metabolic activity may also be potentially useful in the context of patient functional studies, in which some derangement of those mechanisms may make it more difficult to fit neuroimaging data. We have recently demonstrated the use of another variant of our brain model in premature infants undergoing fNIRS monitoring during visual activation. In that case the model's prediction of the cerebral autoregulatory capacity in these infants could explain the sometimes inverted deoxyhaemoglobin response (Hapuarachchi et al., 2016). In the future, we aim to expand this work to other patient populations such as those with traumatic brain injury, and by fitting the data to the model parameters identify potential biomarkers of cerebrovascular disease processes.

In conclusion, despite its limitations the model provides useful information concerning the possible confounding of fNIRS measurements when appropriate input data are recorded. It allows for some attribution between the compartments and may help to identify misleading cases. A full software implementation is freely available (https://github.com/bcmd), so other researchers can use it in conjunction with their own experimental data. With model development ongoing, the ability to better extract relevant meanings from fNIRS and fMRI data will further improve.

## Figures and Tables

**Table 1:** Systemic input variable ranges for simulations. Step inputs in Fig. 3 are from the baseline to the upper bound of each range.

**Input**	**Description**	**Units**	**Baseline**	**Lower**	**Upper**
*P_a_*	Mean arterial pressure	mmHg	100	75	125
$P_{a}{\mathrm{CO}}_{2}$	Partial pressure of ${\mathrm{CO}}_{2}$	mmHg	40	37.5	42.5
$S_{a}O_{2}$	Arterial oxygen saturation	%	96	90	100⁎
*u*	Demand	–	1.0	0.75	1.25
*Flux_y_*	Normalised LD flux	–	1.0	0.75	1.25

⁎ Spontaneous increase in arterial saturation was deemed implausible in typical functional activation experiments, so the baseline value of 96% was used as the upper bound when optimising this parameter for the scenarios in Fig. 5.

## Appendix A Supplementary data

Application 1

Application 2

Application 3

Application 4

## Footnotes

Appendix A Supplementary data associated with this article can be found in the online version at doi:10.1016/j.neuroimage.2016.08.058.
