# Supplementary figures and images for: Modelling confounding effects from extracerebral contamination and systemic factors on functional near-infrared spectroscopy
Source: Neuroimage. 2016 Dec;143:91–105. doi: 10.1016/j.neuroimage.2016.08.058 (PMC5139986; doi:10.1016/j.neuroimage.2016.08.058)

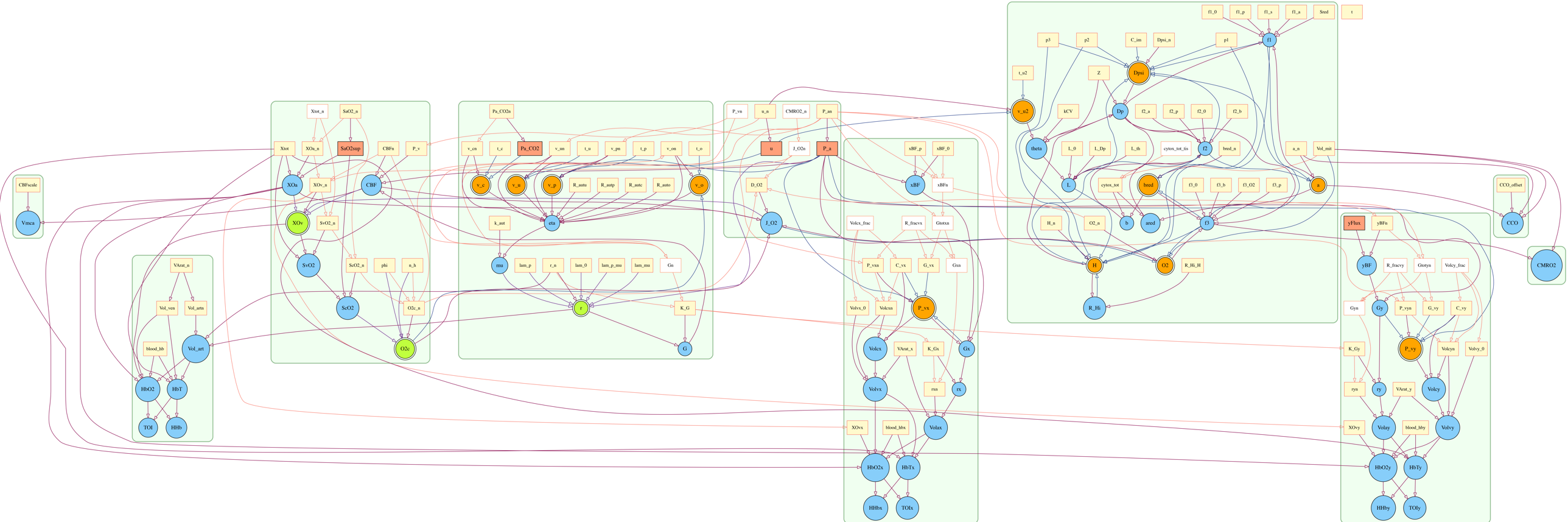

Dependency Graph for model bsx

Supplement: Application 2 [file mmc2.pdf]
