# Supplementary material for: Modelling confounding effects from extracerebral contamination and systemic factors on functional near-infrared spectroscopy
Source: Neuroimage. 2016 Dec;143:91–105. doi: 10.1016/j.neuroimage.2016.08.058 (PMC5139986; doi:10.1016/j.neuroimage.2016.08.058)
Supplement: Application 3 [file mmc3.pdf]

bsx

Generated by BCMD module `doc_latex.py`

June 28, 2016

1 Overview

Extended BrainSignals model, based primarily on the simplified variant B1M2 from BrainSignals Revisited. The metabolic submodel has been modified to improve modelling of functional activation, and an extracerebral blood flow compartment (actually 2 different versions of such a compartment) have been added. The scalp compartments operate in parallel to the cerebral model and do not affect its behaviour.

- 12 differential state variables
- 3 algebraic state variables
- 39 intermediate variables
- 72 independent parameters
- 49 derived parameters
- 5 declared inputs
- 53 default outputs

2 Differential Equations

$$\frac{\mathrm{d}Cu_{A,o}}{\mathrm{d}t} = 4f_3 - 4f_1 \tag{1}$$

$$\frac{\mathrm{d}a_{3,r}}{\mathrm{d}t} = 4f_3 - 4f_3 \tag{2}$$

$$\frac{\mathrm{d}\psi}{\mathrm{d}t} = \frac{p_3\,f_3 + p_1\,f_1 + p_3\,f_3 - L}{C_{im}} \tag{3}$$

$$\frac{\mathrm{d}H^+}{\mathrm{d}t} = \frac{1}{R_{Hi}}\,L - \frac{p_3}{R_{Hi}}\,f_3 - \frac{p_1}{R_{Hi}}\,f_1 - \frac{p_3}{R_{Hi}}\,f_3 \tag{4}$$

$$\frac{\mathrm{d}O_2}{\mathrm{d}t} = \frac{1}{Vol_{mit}}\,J_{O_2} - f_3 \tag{5}$$

$$\frac{\mathrm{d}P_{v,x}}{\mathrm{d}t} = \frac{G_x \, (P_a - P_{v,x}) - P_{v,x} \, G_{v,x}}{C_{v,x}} \tag{6}$$

$$\frac{\mathrm{d}P_{v,y}}{\mathrm{d}t} = \frac{G_y \, \left(P_a - P_{v,y}\right) - P_{v,y} \, G_{v,y}}{C_{v,y}} \tag{7}$$

$$\frac{\mathrm{d}\nu_{CO_2}}{\mathrm{d}t} = \frac{1}{\tau_{CO_2}} \, \left(Pa_{CO_2} - \nu_{CO_2}\right) \tag{8}$$

$$\frac{\mathrm{d}\nu_{O_2}}{\mathrm{d}t} = \frac{1}{\tau_{O_2}} \, \left(O_{2,c} - \nu_{O_2}\right) \tag{9}$$

$$\frac{\mathrm{d}\nu_{P_a}}{\mathrm{d}t} = \frac{1}{\tau_{P_a}} \, \left(P_a - \nu_{P_a}\right) \tag{10}$$

$$\frac{\mathrm{d}\nu_{u}}{\mathrm{d}t} = \frac{1}{\tau_{u}} \, \left(u - \nu_{u}\right) \tag{11}$$

$$\frac{\mathrm{d}\nu_{u,2}}{\mathrm{d}t} = \frac{1}{\tau_{u,2}} \, \left(u - \nu_{u,2}\right) \tag{12}$$

3 Algebraic Equations

$$\phi \, \left(\frac{S_{c,O_2}}{1 - S_{c,O_2}}\right)^{\frac{1}{n_h}} - O_{2,c} = 0 \tag{13}$$

$$\lambda_0 + \frac{\lambda_{P_a}}{P_a} + \lambda_\mu \, \mu + \frac{\lambda_{P_a,\mu} \, \mu}{P_a} - r = 0 \tag{14}$$

$$CBF \, \left(HbO_{2,a} - HbO_{2,v}\right) - J_{O_2} = 0 \tag{15}$$

4 Chemical Reactions

$$\xrightarrow{L} \frac{1}{\mathrm{R_{Hi}}} \, \mathrm{H^+} \tag{16}$$

$$\xrightarrow{J_{O_2}} \frac{1}{\mathrm{Vol_{mit}}} \, \mathrm{O_2} \tag{17}$$

$$\frac{\mathrm{p_3}}{\mathrm{R_{Hi}}}\,\mathrm{H^+} \xrightarrow{f_3} 4\,\mathrm{Cu_{A,o}} + 4\,\mathrm{a_{3,r}} \tag{18}$$

$$4\,\mathrm{Cu_{A,o}} + \frac{\mathrm{p_1}}{\mathrm{R_{Hi}}}\,\mathrm{H^+} \xrightarrow{f_1} \tag{19}$$

$$\mathrm{O_2} + 4\,\mathrm{a_{3,r}} + \frac{\mathrm{p_3}}{\mathrm{R_{Hi}}}\,\mathrm{H^+} \xrightarrow{f_3} \tag{20}$$

5 State Variables

$Cu_{A,o}$
Implementation Name: `a`
Units: mM
Initial value: $Cu_{A,o,n}$
Concentration of oxidised cytochrome c oxidase.

$a_{3,r}$
Implementation Name: `bred`
Units: mM
Initial value: $a_{3,r,n}$
Concentration of reduced cytochrome a_3.

ψ
Implementation Name: `Dpsi`
Units: mV
Initial value: ψ_n
Mitochondrial inner membrane potential. Varies as charge (in the form of protons) is transferred across the membrane capacitance.

H^+
Implementation Name: `H`
Units: mM
Initial value: H_n^+
Mitochondrial proton concentration.

O_2
Implementation Name: `O2`
Units: mM
Initial value: $O_{2,n}$
Mitochondrial oxygen concentration.

$O_{2,c}$
Implementation Name: `O2c`
Units: mM
Initial value: $O_{2,c,n}$
Capillary oxygen concentration.

$P_{v,x}$
Implementation Name: `P_vx`
Units: mmHg
Initial value: $P_{v,x,n}$
Pressure drop over the extracerebral venous compartment in the pressure-based model. This also defines the stored volume. The underlying model for this is essentially a 3-element windkessel.

$P_{v,y}$
Implementation Name: P_vy
Units: mmHg
Initial value: $P_{v,y,n}$
Pressure drop over the extracerebral venous compartment in the flux-based model. This also determines the stored volume. The underlying model for this is essentially a 3-element windkessel.

r
Implementation Name: r
Units: cm
Initial value: r_n
Typical blood vessel radius.

ν_{CO_2}
Implementation Name: v_c
Units: mmHg
Initial value: $\nu_{CO_2,n}$
Filtered carbon dioxide partial pressure.

ν_{O_2}
Implementation Name: v_o
Units: mM
Initial value: $\nu_{O_2,n}$
Filtered capillary oxygen concentration.

ν_{P_a}
Implementation Name: v_p
Units: mmHg
Initial value: $\nu_{P_a,n}$
Filtered arterial blood pressure.

ν_u
Implementation Name: v_u
Units: dimensionless
Initial value: $\nu_{u,n}$
Filtered demand for the haemodynamic submodel.

$\nu_{u,2}$
Implementation Name: v_u2
Units: dimensionless
Initial value: u_n
Filtered demand for the metabolic submodel.

$HbO_{2,v}$
Implementation Name: XOv
Units: mM
Initial value: $HbO_{2,v,n}$
Venous concentration of oxygen bound to haemoglobin.

6 Intermediate Variables

$Cu_{A,r} = CCO_{tot} - Cu_{A,o}$
Implementation Name: ared
Units: mM
Initial value: 0
Concentration of reduced Cu_A.

$a_{3,o} \ = CCO_{tot} - a_{3,r}$
Implementation Name: `b`
Units: mM
Initial value: 0
Concentration of oxidised cytochrome a_3.

$CBF \ = G \ (P_a - P_v)$
Implementation Name: `CBF`
Units: $\mathrm{ml}_{blood}\ \mathrm{ml}_{brain}^{-1}\ \mathrm{s}^{-1}$
Initial value: CBF_n
Cerebral blood flow.

$\Delta oxCCO \ = \Delta oxCCO_{off} + 1000 Vol_{mit} \ (Cu_{A,o} - Cu_{A,o,n})$
Implementation Name: `CCO`
Units: uM
Initial value: 0
Cytochrome c oxidase signal measured by NIRS.

$CMRO_2 \ = f_3 \, Vol_{mit}$
Implementation Name: `CMRO2`
Units: $\mathrm{mM\ s}^{-1}$
Initial value: 0
Rate of cerebral oxygen metabolism.

$\Delta p \ = \psi - Z \ \left(4 + \log 10 \left(H^+\right)\right)$
Implementation Name: `Dp`
Units: mV
Initial value: 0
Proton motive force across the mitochondrial inner membrane.

$\eta \ = R_{P_a} \left(\frac{v_{P_a}}{v_{P_a,n}} - 1\right) + R_{O_2} \left(\frac{v_{O_2}}{v_{O_2,n}} - 1\right) + R_{CO_2} \left(1 - \frac{v_{CO_2}}{v_{CO_2,n}}\right) + R_u \left(1 - \frac{v_u}{v_{u,n}}\right)$
Implementation Name: `eta`
Units: dimensionless
Initial value: 0
Merged autoregulation stimulus.

$f_1 \ = \lambda_{f_1} + \lambda_{f_1,s} \log\left(S_{red}\right) + \lambda_{f_1,a} \log\left(Cu_{A,o}\right) + \lambda_{f_1,p} \, \Delta p$
Implementation Name: `f1`
Units: $\mathrm{mM\ s}^{-1}$
Initial value: 0
Reaction rate for the reduction of Cu_A.

$f_3 \ = \lambda_{f_2} + \lambda_{f_2,a} \log\left(Cu_{A,r}\right) + \lambda_{f_2,b} \log\left(a_{3,o}\right) + \lambda_{f_2,p} \, \Delta p$
Implementation Name: `f2`
Units: $\mathrm{mM\ s}^{-1}$
Initial value: 0
Reaction rate for the reduction of a_3.

$f_3 \ = \lambda_{f_3} + \lambda_{f_3,b} \log\left(a_{3,r}\right) + \lambda_{f_3,O_2} \log\left(O_2\right) + \lambda_{f_3,p} \, \Delta p$
Implementation Name: `f3`
Units: $\mathrm{mM\ s}^{-1}$
Initial value: 0
Reaction rate for the reduction of O_2.

$G \ = K_G \, r^4$
Implementation Name: `G`
Units: $\mathrm{ml}_{blood}\ \mathrm{ml}_{brain}^{-1}\ \mathrm{mmHg}^{-1}\ \mathrm{s}^{-1}$
Initial value: 0
Effective conductance of the whole blood flow compartment.

$G_x = \dfrac{xBF}{P_a - P_{v,x}}$
Implementation Name: `Gx`
Units: $\mathrm{ml}_{blood}\ \mathrm{ml}_{tissue}^{-1}\ \mathrm{mmHg}^{-1}\ \mathrm{s}^{-1}$
Initial value: 0
Extracerebral conductance in the pressure-based model, inferred from blood flow. (This obviously fails as $(P_a - P_{v,x})$ approaches zero.)

$HbO_2 = (V_a\, HbO_{2,a} + V_v\, HbO_{2,v})\ blood_{hb}$
Implementation Name: `HbO2`
Units: uM
Initial value: 0
Oxygenated haemoglobin signal measured by NIRS.

$HbO_{2,x} = (Vol_{a,x}\, HbO_{2,a} + Vol_{v,x}\, HbO_{2,v,x})\ blood_{hb,x}$
Implementation Name: `HbO2x`
Units: uM
Initial value: 0
Extracerebral oxygenated haemoglobin in the pressure-based model.

$HbO_{2,y} = \left(Vol_{a,y}\, HbO_{2,a} + Vol_{v,y}\, HbO_{2,v,y}\right)\ blood_{hb,y}$
Implementation Name: `HbO2y`
Units: uM
Initial value: 0
Extracerebral oxygenated haemoglobin in the flux-based model.

$HbT = (V_a + V_v)\ Hb_{tot}\, blood_{hb}$
Implementation Name: `HbT`
Units: uM
Initial value: 0
Total haemoglobin signal measured by NIRS.

$HbT_x = (Vol_{a,x} + Vol_{v,x})\ Hb_{tot}\, blood_{hb,x}$
Implementation Name: `HbTx`
Units: uM
Initial value: 0
Extracerebral total haemoglobin in the pressure-based model.

$HbT_y = \left(Vol_{a,y} + Vol_{v,y}\right)\ Hb_{tot}\, blood_{hb,y}$
Implementation Name: `HbTy`
Units: uM
Initial value: 0
Extracerebral total haemoglobin in the flux-based model.

$HHb = HbT - HbO_2$
Implementation Name: `HHb`
Units: uM
Initial value: 0
Deoxygenated haemoglobin signal measured by NIRS.

$HHb_x = HbT_x - HbO_{2,x}$
Implementation Name: `HHbx`
Units: uM
Initial value: 0
Extracerebral deoxygenated haemoglobin in the pressure-based model.

$HHb_y = HbT_y - HbO_{2,y}$
Implementation Name: `HHby`
Units: uM
Initial value: 0

Extracerebral deoxygenated haemoglobin in the flux-based model.

$J_{O_2} = \text{fmin}\left(D_{O_2}\,\left(O_{2,c} - O_2\right), CBF\, HbO_{2,a}\right)$
Implementation Name: `J_O2`
Units: mM s^{-1}
Initial value: 0
Oxygen flux from blood to tissue.

$L = \lambda_L + \lambda_{L,\theta}\,\theta + \lambda_{L,p}\,\Delta p$
Implementation Name: `L`
Units: mM s^{-1}
Initial value: 0
Rate of proton return to the mitochondrial matrix.

$\mu = \dfrac{k_{aut}\,\left(\exp\left(\eta\right) - 1\right)}{\exp\left(\eta\right) + 1}$
Implementation Name: `mu`
Units: dimensionless
Initial value: 0
Effective strength of the autoregulation reponse.

$R_{Hi} = \dfrac{R_{Hi,H}}{H^+}$
Implementation Name: `R_Hi`
Units: dimensionless
Initial value: 0
Relative mitochondrial volume for protons, taking into account buffering effect of pH.

$r_x = \left(\dfrac{G_x}{K_{G,x}}\right)^{0.25}$
Implementation Name: `rx`
Units: cm
Initial value: 0
Extracerebral vessel radius in the pressure-based model, inferred from conductance.

$S_{c,O_2} = \dfrac{S_{a,O_2} + S_{v,O_2}}{2}$
Implementation Name: `ScO2`
Units: dimensionless
Initial value: $S_{c,O_2,n}$
Capillary oxygen saturation.

$S_{v,O_2} = \dfrac{HbO_{2,v}}{Hb_{tot}}$
Implementation Name: `SvO2`
Units: dimensionless
Initial value: $S_{v,O_2,n}$
Venous oxygen saturation.

$\theta = kCV\,\left(\Delta p + Z\log10\left(\nu_{u,2}\right) - 90\right)$
Implementation Name: `theta`
Units: dimensionless
Initial value: 0
Driving force Complex V.

$TOI = \dfrac{100HbO_2}{HbT}$
Implementation Name: `TOI`
Units: dimensionless
Initial value: 0
Total oxygenation index.

$TOI_x = \frac{100 HbO_{2,x}}{HbT_x}$
Implementation Name: `TOIx`
Units: dimensionless
Initial value: 0
Extracerebral TOI in the pressure-based model.

$TOI_y = \frac{100 HbO_{2,y}}{HbT_y}$
Implementation Name: `TOIy`
Units: dimensionless
Initial value: 0
Extracerebral TOI in the flux-based model.

$V_{mca} = CBF\, CBFscale$
Implementation Name: `Vmca`
Units: cm s^{-1}
Initial value: 0
Blood velocity in the middle cerebral artery.

$V_a = V_{a,n} \left(\frac{r}{r_n}\right)^2$
Implementation Name: `Vol_art`
Units: dimensionless
Initial value: 0
Relative arterial blood volume.

$Vol_{a,x} = \frac{\left(\frac{r_x}{r_{x,n}}\right)^2}{1 + VA_{rat,x}}$
Implementation Name: `Volax`
Units: dimensionless
Initial value: 0
Relative extracerebral arterial volume in the pressure-based model, compared to the 'normal' value. Both arterial and venous volumes may vary, but we assume that the balance when all values are normal produces the normal venous/arterial ratio.

$Vol_{c,x} = P_{v,x}\, C_{v,x}$
Implementation Name: `Volcx`
Units: $\mathrm{ml}_{blood}\ \mathrm{ml}_{tissue}^{-1}$
Initial value: $Vol_{c,x,n}$
Volume contribution from venous compliance in the pressure-based model. (This is effectively dimensionless, but we specify units to emphasise scaling consistency with the compliance parameter.)

$Vol_{c,y} = P_{v,y}\, C_{v,y}$
Implementation Name: `Volcy`
Units: $\mathrm{ml}_{blood}\ \mathrm{ml}_{tissue}^{-1}$
Initial value: $Vol_{c,y,n}$
Volume contribution from venous compliance in the flux-based model. (This is effectively dimensionless, but we specify units to emphasise scaling consistency with the compliance parameter.)

$Vol_{v,x} = \frac{\left(Vol_{v,x,0} + \frac{Vol_{c,x}}{Vol_{c,x,n}}\right) VA_{rat,x}}{1 + VA_{rat,x}}$
Implementation Name: `Volvx`
Units: dimensionless
Initial value: 0
Relative extracerebral venous volume in the pressure-based model. We assume this consists

of a constant base volume plus a fraction that varies with compliance, scaled such that at normality the total is 1.

$$Vol_{v,y} = \frac{\left(Vol_{v,y,0} + \frac{Vol_{c,y}}{Vol_{c,y,n}}\right) VA_{rat,y}}{1 + VA_{rat,y}}$$

Implementation Name: `Volvy`
Units: dimensionless
Initial value: 0
Relative extracerebral venous volume in the flux-based model. We assume this consists of a constant base volume plus a fraction that varies with compliance, scaled such that at normality the total is 1.

7 Parameters

$Cu_{A,o,n}$
Implementation Name: `a_n`
Units: mM
Initial value: 0.06567
Normal concentration of oxidised cytochrome c oxidase.

$blood_{hb}$
Implementation Name: `blood_hb`
Units: dimensionless
Initial value: 10.00
Factor to convert model haemoglobin concentration to instrumental units. Scales for blood fraction of brain volume, mM to μM, and number of binding sites.

$blood_{hb,x}$
Implementation Name: `blood_hbx`
Units: dimensionless
Initial value: 10.00
Haemoglobin fraction in scalp in the pressure-based model. As with the cerebral fraction, this term combines scaling for units and haemoglobin binding site with a blood fraction estimate.

$blood_{hb,y}$
Implementation Name: `blood_hby`
Units: dimensionless
Initial value: 10.00
Haemoglobin fraction in scalp in the flux-based model. As with the cerebral fraction, this term combines scaling for units and haemoglobin binding site with a blood fraction estimate.

$a_{3,r,n}$
Implementation Name: `bred_n`
Units: mM
Initial value: 0.001408
Normal concentration of reduced cytochrome a3.

C_{im}
Implementation Name: `C_im`
Units: mM mV^{-1}
Initial value: 0.00675
Capacitance of the mitochondrial inner membrane.

CBF_n
Implementation Name: `CBFn`

Units: ml_{blood} ml_{brain}^{-1} s^{-1}
Initial value: 0.0125
Normal cerebral blood flow.

$CBFscale$
Implementation Name: `CBFscale`
Units: cm
Initial value: 5000
Scale constant relating blood flow to arterial velocity.

$\Delta oxCCO_{off}$
Implementation Name: `CCO_offset`
Units: uM
Initial value: 0
Signal offset for the NIRS CCO measurement.

$CMRO_{2,n}$
Implementation Name: `CMRO2_n`
Units: mM s^{-1}
Initial value: 0.034
Normal metabolic rate of oxygen consumption.

CCO_{tis}
Implementation Name: `cytox_tot_tis`
Units: mM
Initial value: 0.0055
Concentration of cytochrome c oxidase in tissue.

ψ_n
Implementation Name: `Dpsi_n`
Units: mV
Initial value: 145
Normal mitochondrial inner membrane potential.

λ_{f_1}
Implementation Name: `f1_0`
Units: mM s^{-1}
Initial value: 4.336
Fitted intercept for the linear model for f_1.

$\lambda_{f_1,a}$
Implementation Name: `f1_a`
Units: mM s^{-1}
Initial value: 0.7146
Fitted linear dependence of f_1 on logarithm of $Cu_{A,ox}$.

$\lambda_{f_1,p}$
Implementation Name: `f1_p`
Units: mM s^{-1} mV^{-1}
Initial value: -0.01117
Fitted linear dependence of f_1 on Δp.

$\lambda_{f_1,s}$
Implementation Name: `f1_s`
Units: mM s^{-1}
Initial value: 1
Fitted linear dependence of f_1 on logarithm of relative supply.

λ_{f_2}
Implementation Name: `f2_0`
Units: mM s^{-1}

Initial value: 10.41
Fitted intercept for the linear model for f_2.

$\lambda_{f_2,a}$
Implementation Name: f2_a
Units: mM s^{-1}
Initial value: -0.1137
Fitted linear dependence of f_2 on logarithm of $Cu_{A,red}$.

$\lambda_{f_2,b}$
Implementation Name: f2_b
Units: mM s^{-1}
Initial value: 3.188
Fitted linear dependence of f_2 on logarithm of $a_{3,ox}$.

$\lambda_{f_2,p}$
Implementation Name: f2_p
Units: mM s^{-1} mV^{-1}
Initial value: -0.01390
Fitted linear dependence of f_2 on Δp.

λ_{f_3}
Implementation Name: f3_0
Units: mM s^{-1}
Initial value: 9.282
Fitted intercept for the linear model for f_3.

$\lambda_{f_3,b}$
Implementation Name: f3_b
Units: mM s^{-1}
Initial value: 0.2324
Fitted linear dependence of f_3 on logarithm of $a_{3,red}$.

λ_{f_3,O_2}
Implementation Name: f3_O2
Units: mM s^{-1}
Initial value: 0.2918
Fitted linear dependence of f_3 on logarithm of O_2.

$\lambda_{f_3,p}$
Implementation Name: f3_p
Units: mM s^{-1} mV^{-1}
Initial value: -0.03654
Fitted linear dependence of f_3 on Δp.

H_n^+
Implementation Name: H_n
Units: mM
Initial value: 0.00003981
Normal mitochondrial proton concentration.

k_{aut}
Implementation Name: k_aut
Units: dimensionless
Initial value: 1
Overall functioning of autoregulatory response.

kCV
Implementation Name: kCV
Units: mV^{-1}

Initial value: 0.02047339
Factor relating the Complex V driving force to the membrane potential and demand.

λ_L
Implementation Name: L_0
Units: mM s^{-1}
Initial value: -14.86
Fitted intercept for the linear model for L.

$\lambda_{L,p}$
Implementation Name: L_Dp
Units: mM s^{-1} mV^{-1}
Initial value: 0.09440
Fitted linear dependence of L on Δp.

$\lambda_{L,\theta}$
Implementation Name: L_th
Units: mM s^{-1}
Initial value: 5.653
Fitted linear dependence of L on θ.

λ_0
Implementation Name: lam_0
Units: cm
Initial value: 0.02507
Intercept of the fitted linear model for blood vessel radius.

λ_μ
Implementation Name: lam_mu
Units: cm
Initial value: -0.0004422
Fitted linear dependence of blood vessel radius on autoregulatory stimuli.

λ_{P_a}
Implementation Name: lam_p
Units: cm mmHg
Initial value: -0.6327
Fitted linear dependence of blood vessel radius on reciprocal of blood pressure.

$\lambda_{P_a,\mu}$
Implementation Name: lam_p_mu
Units: cm mmHg
Initial value: -0.5286
Fitted joint dependence of blood vessel radius on autoregulatory stimuli and reciprocal of blood pressure.

n_h
Implementation Name: n_h
Units: dimensionless
Initial value: 2.5
Hill coefficient for oxygen dissociation from haemoglobin.

$O_{2,n}$
Implementation Name: O2_n
Units: mM
Initial value: 0.024
Normal mitochondrial oxygen concentration.

p_1
Implementation Name: p1
Units: dimensionless

Initial value: 12
Proton cost of the reaction reducing Cu_A.

p_3

Implementation Name: p2
Units: dimensionless
Initial value: 4
Proton cost of the reaction reducing a_3.

p_3

Implementation Name: p3
Units: dimensionless
Initial value: 4
Proton cost of the reaction reducing O_2.

$P_{a,n}$

Implementation Name: P_an
Units: mmHg
Initial value: 100
Normal arterial blood pressure.

$P_{v,n}$

Implementation Name: P_vn
Units: mmHg
Initial value: 4
Normal venous blood pressure.

$Pa_{CO_{2,n}}$

Implementation Name: Pa_CO2n
Units: mmHg
Initial value: 40
Normal arterial partial pressure of carbon dioxide.

ϕ

Implementation Name: phi
Units: mM
Initial value: 0.036
Oxygen concentration at half-maximal saturation.

R_{CO_2}

Implementation Name: R_autc
Units: dimensionless
Initial value: 2.2
Autoregulatory reactivity to carbon dioxide.

R_{O_2}

Implementation Name: R_auto
Units: dimensionless
Initial value: 1.5
Autoregulatory reactivity to oxygen.

R_{P_a}

Implementation Name: R_autp
Units: dimensionless
Initial value: 4
Autoregulatory reactivity to blood pressure.

R_u

Implementation Name: R_autu
Units: dimensionless

Initial value: 0.5
Autoregulatory reactivity to demand.

$R_{frac,v,x}$
Implementation Name: R_fracvx
Units: dimensionless
Initial value: 0.1
Fraction of normal total resistance that resides in the venous compartment in the pressure-based model. We expect this to be small in general.

$R_{frac,v,y}$
Implementation Name: R_fracvy
Units: dimensionless
Initial value: 0.1
Fraction of normal total resistance that resides in the venous compartment in the flux-based model. We expect this to be small in general.

$R_{Hi,H}$
Implementation Name: R_Hi_H
Units: mM
Initial value: 9.565483
Proton buffering factor.

r_n
Implementation Name: r_n
Units: cm
Initial value: 0.0187
Normal effective blood vessel radius.

$S_{a,O_2,n}$
Implementation Name: SaO2_n
Units: dimensionless
Initial value: 0.96
Normal arterial oxygen saturation.

S_{red}
Implementation Name: Sred
Units: not defined
Initial value: 1
Parameter representing the relative supply of reducing substrate. This is normally 1, and the dependency in f1 is logarithmic, so this term normally disappears, but we may adjust it later to investigate supply restrictions.

t
Implementation Name: t
Units: s
Initial value: 0
Time over which the system evolves.

τ_{CO_2}
Implementation Name: t_c
Units: s
Initial value: 5
Filter time constant for stimulus effect of carbon dioxide.

τ_{O_2}
Implementation Name: t_o
Units: s
Initial value: 20
Filter time constant for stimulus effect of capillary oxygen.

τ_{P_a}

Implementation Name: `t_p`
Units: s
Initial value: 5
Filter time constant for stimulus effect of blood pressure.

τ_u

Implementation Name: `t_u`
Units: s
Initial value: 0.5
Filter time constant for stimulus effect of demand.

$\tau_{u,2}$

Implementation Name: `t_u2`
Units: s
Initial value: 0.5
Filter time constant for metabolic effect of demand.

u_n

Implementation Name: `u_n`
Units: dimensionless
Initial value: 1
Normal demand.

$VArat_n$

Implementation Name: `VArat_n`
Units: dimensionless
Initial value: 3
Normal volume ratio of veins to arteries in brain tissue.

$VA_{rat,x}$

Implementation Name: `VArat_x`
Units: dimensionless
Initial value: 3
'Normal' ratio of extracerebral veins to arteries in the pressure-based model.

$VA_{rat,y}$

Implementation Name: `VArat_y`
Units: dimensionless
Initial value: 3
'Normal' ratio of extracerebral veins to arteries in the flux-based model.

Vol_{mit}

Implementation Name: `Vol_mit`
Units: dimensionless
Initial value: 0.067
Fraction of brain tissue volume that is mitochondria.

$Vol_{c,x,frac}$

Implementation Name: `Volcx_frac`
Units: dimensionless
Initial value: 0.1
Fraction of normal venous volume that is due to the venous compliance in the pressure-based model. This volume is subject to variation when the system is not at baseline, whereas the remainder is constant.

$Vol_{c,y,frac}$

Implementation Name: `Volcy_frac`
Units: dimensionless
Initial value: 0.1

Fraction of normal venous volume that is due to the venous compliance in the flux-based model. This volume is subject to variation when the system is not at baseline, whereas the remainder is constant.

λ_{F_x}
Implementation Name: xBF_0
Units: ml_{blood} 100 ml_{tissue}^{-1} min^{-1}
Initial value: -13.4
Intercept of blood flow linear model in the pressure-based model. Fitted from data in Gagnon et al (2014).

$\lambda_{F_{x,p}}$
Implementation Name: xBF_p
Units: ml_{blood} 100 ml_{tissue}^{-1} min^{-1} $mmHg^{-1}$
Initial value: 0.48
Dependence of extracerebral blood flow on P_a in the pressure-based model. Fitted from data in Gagnon et al (2014).

Hb_{tot}
Implementation Name: Xtot
Units: mM
Initial value: 9.1
Total concentration of haemoglobin O_2 binding sites in blood (4 times haemoglobin concentration).

$Hb_{tot,n}$
Implementation Name: Xtot_n
Units: mM
Initial value: 9.1
Normal total concentration of haemoglobin O_2 binding sites in blood (4 times haemoglobin concentration).

$yFlux$
Implementation Name: yFlux
Units: not defined
Initial value: 1
Superficial blood "flux" from Laser Doppler or equivalent instrumentation. This does not provide an absolute measurement, but instead is relative to some baseline condition which is (of course) unknown and hence has a basically arbitrary scale and offset. We assume that this has been factored out such that yFlux is equal to 1 at yBFn.

Z
Implementation Name: Z
Units: mV
Initial value: 59.028
Proportionality constant in calculation of driving forces due to concentration differences. Defined as RT/F, where F is Faraday's constant, R the ideal gas constant and T the absolute temperature.

8 Derived Parameters

$C_{v,x}$
Implementation Name: C_vx
Units: ml_{blood} ml_{tissue}^{-1} $mmHg^{-1}$
Initial value: $\frac{0.00051}{Vol_{c,x,frac}}$
Compliance of the venous compartment in the pressure-based model. Decent estimates for

the scalp are elusive, but we start with a ballpark for peripheral veins in general from Olsen and Länne (1998). They find lower body compliance of 0.051 ml per 100 ml per mmHg in young subjects. We scale this to account for the compliant volume fraction and the units change.

$C_{v,y}$
Implementation Name: `C_vy`
Units: $\text{ml}_{blood}\ \text{ml}_{tissue}^{-1}\ \text{mmHg}^{-1}$
Initial value: $\frac{0.00051}{Vol_{c,y,frac}}$
Compliance of the venous compartment in the flux-based model. Decent estimates for the scalp are elusive, but we start with a ballpark for peripheral veins in general from Olsen and Länne (1998). They find lower body compliance of 0.051 ml per 100 ml per mmHg in young subjects. We scale this to account for the compliant volume fraction and the units change.

CCO_{tot}
Implementation Name: `cytox_tot`
Units: mM
Initial value: $\frac{CCO_{tis}}{Vol_{mit}}$
Concentration of cytochrome c oxidase in mitochondria.

D_{O_2}
Implementation Name: `D_O2`
Units: s^{-1}
Initial value: $\frac{J_{O_{2,n}}}{O_{2,c,n} - O_{2,n}}$
Diffusion rate for oxygen between capillaries and mitochondria.

$G_{v,x}$
Implementation Name: `G_vx`
Units: $\text{ml}_{blood}\ \text{ml}_{tissue}^{-1}\ \text{mmHg}^{-1}\ \text{s}^{-1}$
Initial value: $\frac{G_{tot,x,n}}{R_{frac,v,x}}$
Conductance of the venous compartment in the pressure-based model. This is assumed constant.

$G_{v,y}$
Implementation Name: `G_vy`
Units: $\text{ml}_{blood}\ \text{ml}_{tissue}^{-1}\ \text{mmHg}^{-1}\ \text{s}^{-1}$
Initial value: $\frac{G_{tot,y,n}}{R_{frac,v,y}}$
Conductance of the venous compartment in the flux-based model. This is assumed constant.

G_n
Implementation Name: `Gn`
Units: $\text{ml}_{blood}\ \text{ml}_{brain}^{-1}\ \text{mmHg}^{-1}\ \text{s}^{-1}$
Initial value: $\frac{CBF_n}{P_{a,n} - P_{v,n}}$
Normal blood vessel conductance.

$G_{tot,x,n}$
Implementation Name: `Gtotxn`
Units: $\text{ml}_{blood}\ \text{ml}_{tissue}^{-1}\ \text{mmHg}^{-1}\ \text{s}^{-1}$
Initial value: $\frac{xBF_n}{P_{a,n}}$
'Normal' total extracerebral conductance in the pressure-based model, i.e. conductance for normal flow at normal systemic and venous pressures.

$G_{tot,y,n}$

Implementation Name: Gtotyn
Units: $\text{ml}_{blood}\ \text{ml}_{tissue}^{-1}\ \text{mmHg}^{-1}\ \text{s}^{-1}$
Initial value: $\frac{yBF_n}{P_{a,n}}$
'Normal' total extracerebral conductance in the flux-based model, i.e. conductance for normal flow at normal systemic and venous pressures.

$G_{x,n}$

Implementation Name: Gxn
Units: $\text{ml}_{blood}\ \text{ml}_{tissue}^{-1}\ \text{mmHg}^{-1}\ \text{s}^{-1}$
Initial value: $\frac{G_{tot,x,n}}{1 - R_{frac,v,x}}$
Normal conductance of the arterial compartment in the pressure-based model.

$G_y = \frac{yBF}{P_a}$

Implementation Name: Gy
Units: $\text{ml}_{blood}\ \text{ml}_{tissue}^{-1}\ \text{mmHg}^{-1}\ \text{s}^{-1}$
Initial value: 0
Extracerebral conductance in the flux-based model, inferred from flow.

$G_{y,n}$

Implementation Name: Gyn
Units: $\text{ml}_{blood}\ \text{ml}_{tissue}^{-1}\ \text{mmHg}^{-1}\ \text{s}^{-1}$
Initial value: $\frac{G_{tot,y,n}}{1 - R_{frac,v,y}}$
Normal conductance of the arterial compartment in the flux-based model.

$J_{O_{2,n}}$

Implementation Name: J_O2n
Units: mM s^{-1}
Initial value: $CMRO_{2,n}$
Normal oxygen flux from blood to tissue.

K_G

Implementation Name: K_G
Units: $\text{ml}_{blood}\ \text{ml}_{brain}^{-1}\ \text{mmHg}^{-1}\ \text{s}^{-1}\ \text{cm}^{-4}$
Initial value: $\frac{G_n}{r_n^4}$
Proportionality constant in Poiseuille relation for conductance.

$K_{G,x}$

Implementation Name: K_Gx
Units: $\text{ml}_{blood}\ \text{ml}_{tissue}^{-1}\ \text{mmHg}^{-1}\ \text{s}^{-1}\ \text{cm}^{-4}$
Initial value: K_G
Conductance/flow proportionality constant for extracerebral vessels in the pressure-based model. Since we have no basis for setting this, for the moment we just assume it matches the internal value.

$K_{G,y}$

Implementation Name: K_Gy
Units: $\text{ml}_{blood}\ \text{ml}_{tissue}^{-1}\ \text{mmHg}^{-1}\ \text{s}^{-1}\ \text{cm}^{-4}$
Initial value: K_G
Conductance/flow proportionality constant for extracerebral vessels in the flux-based model. Since we have no basis for setting this, for the moment we just assume it matches the internal value.

$O_{2,c,n}$

Implementation Name: `O2c_n`
Units: mM
Initial value: $\phi \left(\frac{S_{c,O_2,n}}{1 - S_{c,O_2,n}} \right)^{\frac{1}{n_h}}$
Normal capillary oxygen concentration.

P_a

Implementation Name: `P_a`
Units: mmHg
Initial value: $P_{a,n}$
Mean arterial blood pressure.

P_v

Implementation Name: `P_v`
Units: mmHg
Initial value: $P_{v,n}$
Venous blood pressure.

$P_{v,x,n}$

Implementation Name: `P_vxn`
Units: mmHg
Initial value: $R_{frac,v,x}\, P_{a,n}$
Normal venous pressure in the pressure-based model. At normal arterial pressure and normal conductance the venous compliance should be steady and the total resistance should divide according to $R_{frac,v,x}$.

$P_{v,y,n}$

Implementation Name: `P_vyn`
Units: mmHg
Initial value: $R_{frac,v,y}\, P_{a,n}$
Normal venous pressure in the flux-based model. At normal arterial pressure and normal conductance the venous compliance should be steady and the total resistance should divide according to $R_{frac,v,y}$.

Pa_{CO_2}

Implementation Name: `Pa_CO2`
Units: mmHg
Initial value: $Pa_{CO_{2,n}}$
Arterial partial pressure of carbon dioxide.

$r_{x,n}$

Implementation Name: `rxn`
Units: cm
Initial value: $\left(\frac{G_{x,n}}{K_{G,x}} \right)^{0.25}$
'Normal' extracerebral vessel radius in the pressure-based model. This is the value implied by normal conductance of the arterial compartment. (Venous compartment is assumed not to contribute to the flow variability.)

$r_y = \left(\frac{G_y}{K_{G,y}} \right)^{0.25}$

Implementation Name: `ry`
Units: cm
Initial value: 0
Extracerebral vessel radius in the flux-based model, inferred from conductance. We assume that the proportionality constant here is the same as for the cerebral model. This probably is not the case, but we have no basis for making a different estimate; this at least has the benefit of consistency.

$r_{y,n}$

Implementation Name: `ryn`
Units: cm
Initial value: $\left(\frac{G_{y,n}}{K_{G,y}}\right)^{0.25}$
'Normal' extracerebral vessel radius in the flux-based model. This is the value implied by the normal conductance of the arterial compartment. (The venous compartment is assumed not to contribute to the flow variability.)

S_{a,O_2}

Implementation Name: `SaO2sup`
Units: dimensionless
Initial value: $S_{a,O_2,n}$
Arterial oxygen saturation.

$S_{c,O_2,n}$

Implementation Name: `ScO2_n`
Units: dimensionless
Initial value: $\frac{S_{a,O_2,n} + S_{v,O_2,n}}{2}$
Normal capillary oxygen saturation.

$S_{v,O_2,n}$

Implementation Name: `SvO2_n`
Units: dimensionless
Initial value: $\frac{HbO_{2,v,n}}{Hb_{tot,n}}$
Normal venous oxygen saturation.

u

Implementation Name: `u`
Units: dimensionless
Initial value: u_n
Parameter indicating metabolic demand.

$v_{CO_2,n}$

Implementation Name: `v_cn`
Units: mmHg
Initial value: $Pa_{CO_{2,n}}$
Normal filtered carbon dioxide partial pressure.

$v_{O_2,n}$

Implementation Name: `v_on`
Units: mM
Initial value: $O_{2,c,n}$
Normal filtered capillary oxygen concentration.

$v_{P_a,n}$

Implementation Name: `v_pn`
Units: mmHg
Initial value: $P_{a,n}$
Normal filtered arterial blood pressure.

$v_{u,n}$

Implementation Name: `v_un`
Units: dimensionless
Initial value: u_n
Normal filtered demand.

$$V_{a,n} = \frac{1}{1 + VArat_n}$$

Implementation Name: `Vol_artn`
Units: dimensionless
Initial value: 0
Normal relative arterial blood volume.

$V_v = \dfrac{VArat_n}{1 + VArat_n}$
Implementation Name: `Vol_ven`
Units: dimensionless
Initial value: 0
Relative venous blood volume.

$Vol_{a,y} = \dfrac{\left(\frac{r_y}{r_{y,n}}\right)^2}{1 + VA_{rat,y}}$
Implementation Name: `Volay`
Units: dimensionless
Initial value: 0
Relative extracerebral arterial volume in the flux-based model, compared to the ‘normal’ value. Both arterial and venous volumes may vary, but we assume that the balance when all values are normal produces the normal venous/arterial ratio.

$Vol_{c,x,n}$
Implementation Name: `Volcxn`
Units: $\text{ml}_{blood}\ \text{ml}_{tissue}^{-1}$
Initial value: $\dfrac{C_{v,x}\ P_{v,x,n}}{Vol_{c,x,frac}}$
Normal compliance-dependent venous volume in the pressure-based model. (This is effectively dimensionless, but we specify units to emphasise scaling consistency with the compliance parameter.)

$Vol_{c,y,n}$
Implementation Name: `Volcyn`
Units: $\text{ml}_{blood}\ \text{ml}_{tissue}^{-1}$
Initial value: $\dfrac{C_{v,y}\ P_{v,y,n}}{Vol_{c,y,frac}}$
Normal compliance-dependent venous volume in the flux-based model. (This is effectively dimensionless, but we specify units to emphasise scaling consistency with the compliance parameter.)

$Vol_{v,x,0}$
Implementation Name: `Volvx_0`
Units: dimensionless
Initial value: $1 - Vol_{c,x,frac}$
Compliance-independent (ie, constant) contribution to venous volume in the pressure-based model.

$Vol_{v,y,0}$
Implementation Name: `Volvy_0`
Units: dimensionless
Initial value: $1 - Vol_{c,y,frac}$
Compliance-independent (ie, constant) contribution to venous volume in the flux-based model.

$xBF = \dfrac{P_a\ \lambda_{F_{x,p}} + \lambda_{F_x}}{6000}$
Implementation Name: `xBF`
Units: $\text{ml}_{blood}\ \text{ml}_{tissue}^{-1}\ \text{s}^{-1}$
Initial value: 0
Extracerebral blood flow in the pressure-based model. At present we assume that this is

dependent solely on arterial pressure, with a simple linear model fitted from data in Gagnon et al (2014). The denominator of 6000 is for unit conversion.

xBF_n
Implementation Name: `xBFn`
Units: $\mathrm{ml}_{blood}\ \mathrm{ml}_{tissue}^{-1}\ \mathrm{s}^{-1}$
Initial value: $\dfrac{P_{a,n}\,\lambda_{F_{x,p}} - \lambda_{F_x}}{6000}$
'Normal' extracerebral blood flow in the pressure-based model, i.e. flow at normal pressure.

$HbO_{2,a} = Hb_{tot}\,S_{a,O_2}$
Implementation Name: `XOa`
Units: mM
Initial value: $HbO_{2,a,n}$
Arterial concentration of oxygen bound to haemoglobin.

$HbO_{2,a,n}$
Implementation Name: `XOa_n`
Units: mM
Initial value: $Hb_{tot,n}\,S_{a,O_2,n}$
Normal arterial concentration of oxygen bound to haemoglobin.

$HbO_{2,v,n}$
Implementation Name: `XOv_n`
Units: mM
Initial value: $\dfrac{CBF_n\,HbO_{2,a,n} - J_{O_{2,n}}}{CBF_n}$
Normal venous concentration of oxygen bound to haemoglobin.

$HbO_{2,v,x}$
Implementation Name: `XOvx`
Units: mM
Initial value: $HbO_{2,v,n}$
Venous concentration of oxygen bound to haemoglobin in the extracerebral tissue in the pressure-based model. Since we do not model oxygen consumption in the extracerebral model, we assume this is constant and currently just import the baseline value from the cerebral model.

$HbO_{2,v,y}$
Implementation Name: `XOvy`
Units: mM
Initial value: $HbO_{2,v,n}$
Venous concentration of oxygen bound to haemoglobin in the extracerebral tissue in the flux-based model. Since we do not model oxygen consumption in the extracerebral model, we assume this is constant and currently just import the baseline value from the cerebral model.

$yBF = yFlux\,yBF_n$
Implementation Name: `yBF`
Units: $\mathrm{ml}_{blood}\ \mathrm{ml}_{tissue}^{-1}\ \mathrm{s}^{-1}$
Initial value: 0
Extracerebral blood flow in the flux-based model. For the moment we assume it can be estimated directly from the flux.

yBF_n
Implementation Name: `yBFn`
Units: $\mathrm{ml}_{blood}\ \mathrm{ml}_{tissue}^{-1}\ \mathrm{s}^{-1}$
Initial value: xBF_n
'Normal' extracerebral blood flow in the flux-based model, i.e. when normalised flux = 1. At the moment we use the same value as the pressure model at normal pressue.
