# Supplementary material for: Modelling confounding effects from extracerebral contamination and systemic factors on functional near-infrared spectroscopy
Source: Neuroimage. 2016 Dec;143:91–105. doi: 10.1016/j.neuroimage.2016.08.058 (PMC5139986; doi:10.1016/j.neuroimage.2016.08.058)
Supplement: Application 4 [file mmc4.pdf]

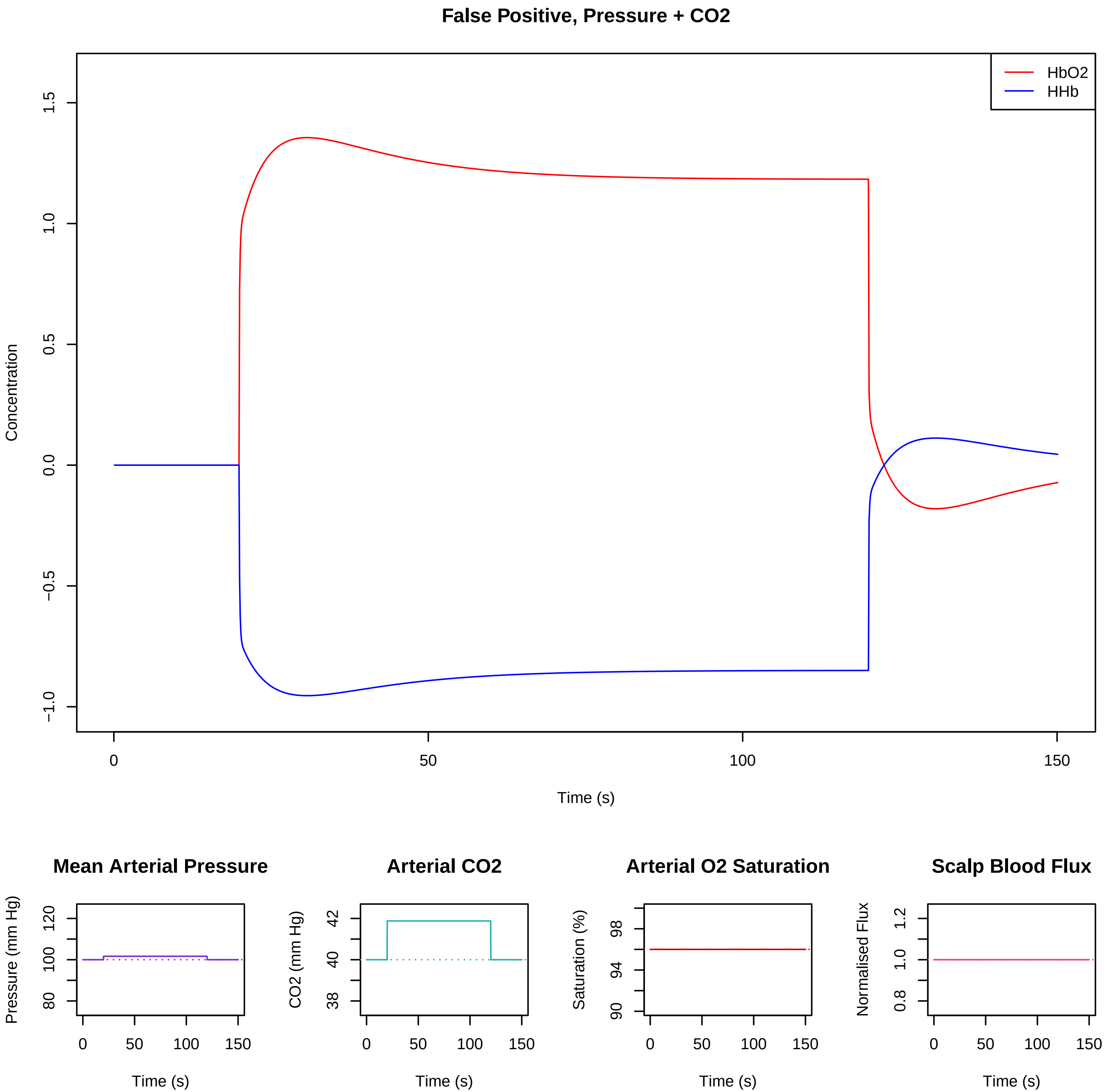

False Positive, Pressure + CO2
HbO2
HHb
Concentration
1.5
1.0
0.5
0.0
−0.5
−1.0
0
50
100
150
Time (s)
Mean Arterial Pressure
Pressure (mm Hg)
120
100
80
Arterial CO2
CO2 (mm Hg)
42
40
38
Arterial O2 Saturation
Saturation (%)
98
94
90
Scalp Blood Flux
Normalised Flux
1.2
1.0
0.8

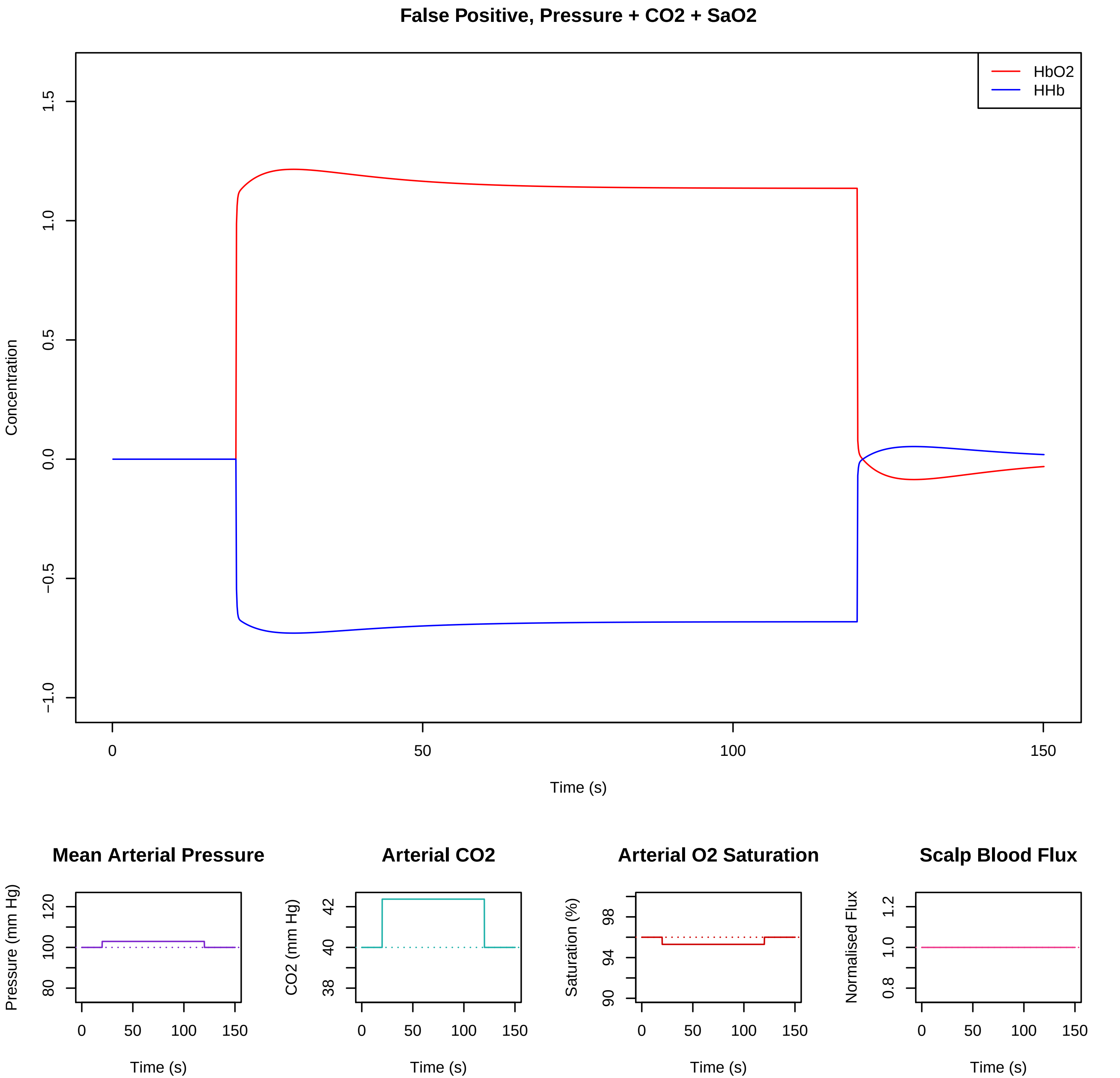

False Positive, Pressure + CO2 + SaO2
HbO2
HHb
Concentration
1.5
1.0
0.5
0.0
−0.5
−1.0
0
50
100
150
Time (s)
Mean Arterial Pressure
Pressure (mm Hg)
80
100
120
Arterial CO2
CO2 (mm Hg)
38
40
42
Arterial O2 Saturation
Saturation (%)
90
94
98
Scalp Blood Flux
Normalised Flux
0.8
1.0
1.2

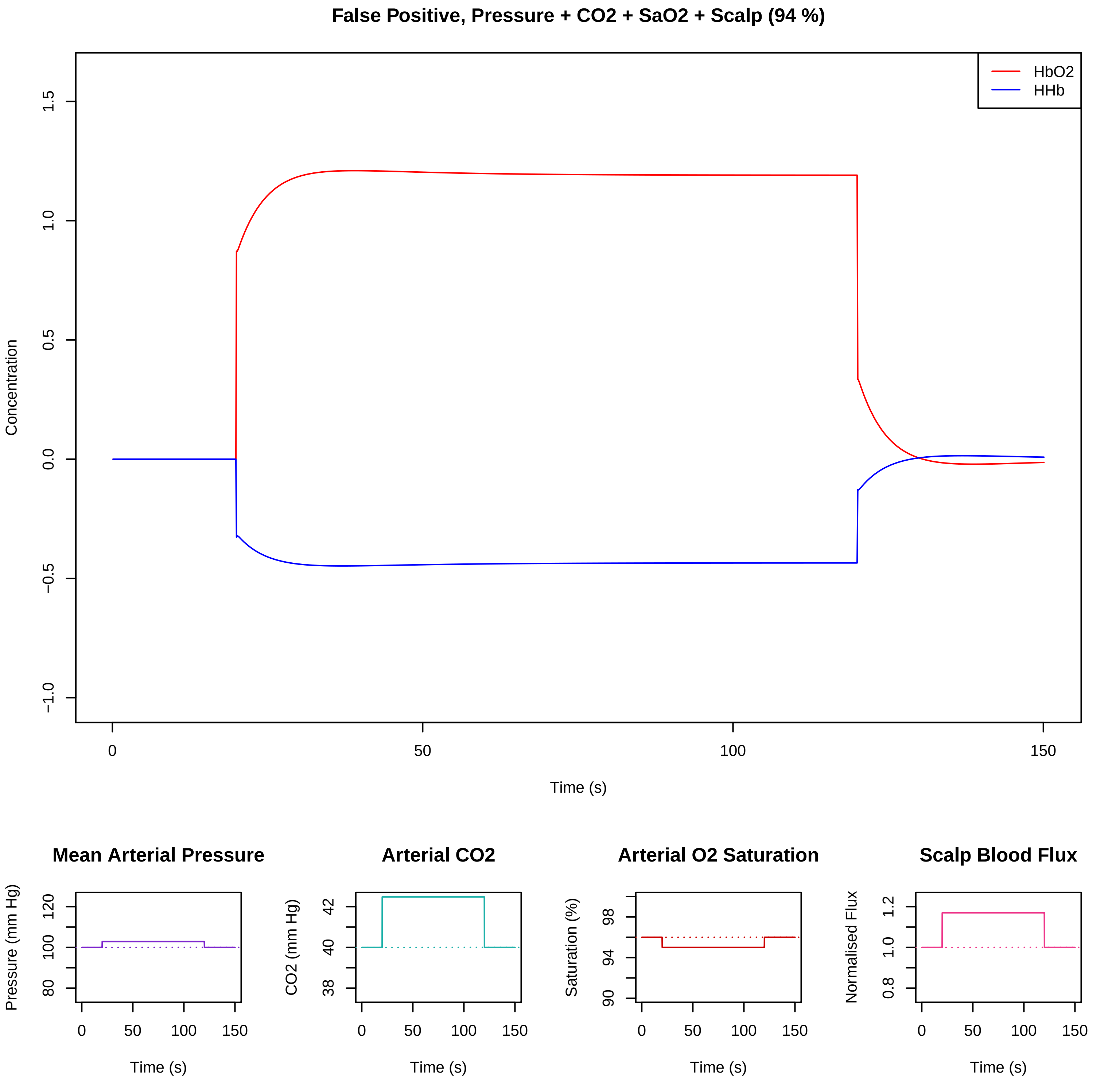
False Positive, Pressure + CO2 + SaO2 + Scalp (94 %)
HbO2
HHb
Concentration
Time (s)
Mean Arterial Pressure
Pressure (mm Hg)
Arterial CO2
CO2 (mm Hg)
Arterial O2 Saturation
Saturation (%)
Scalp Blood Flux
Normalised Flux

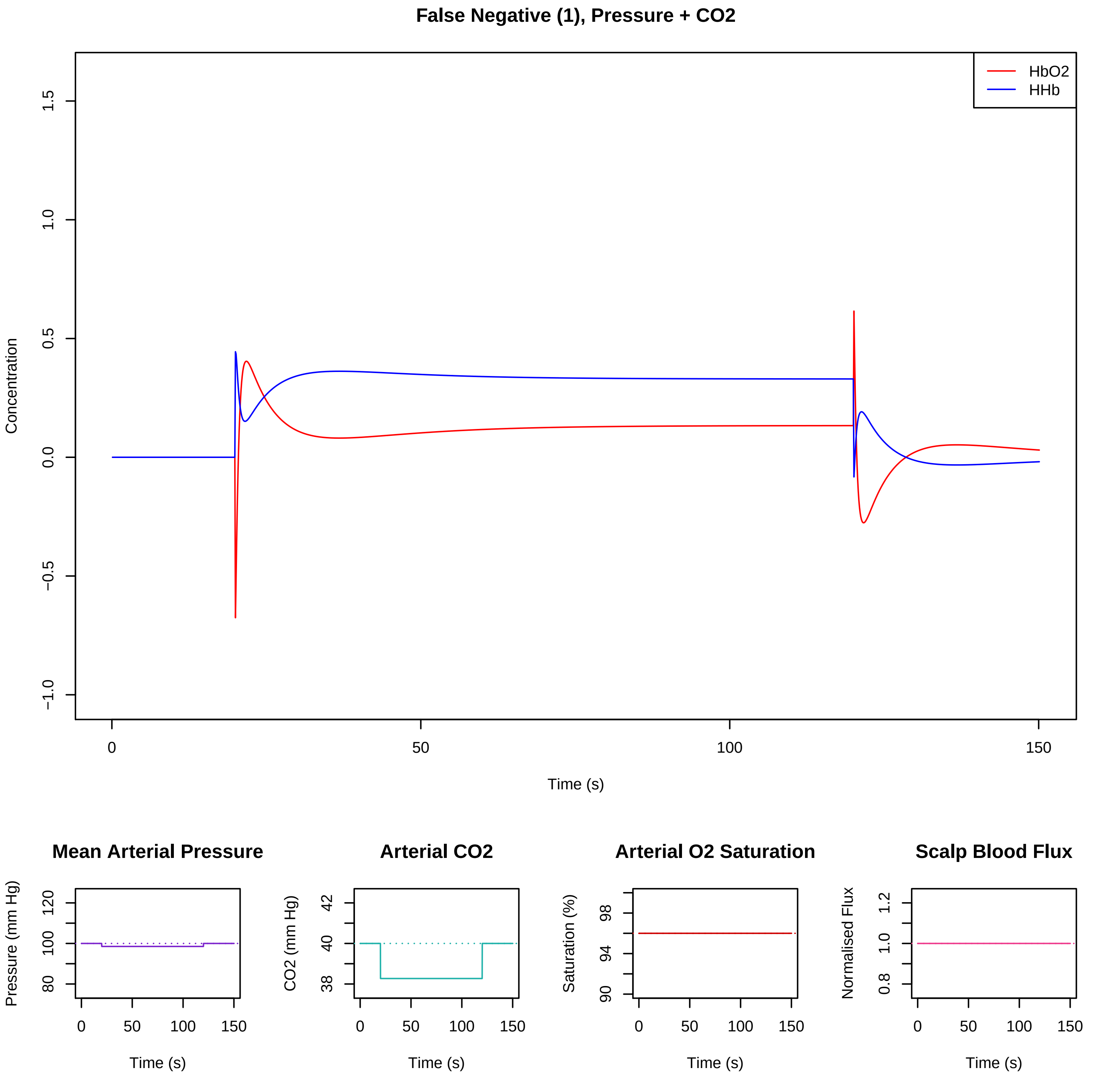
False Negative (1), Pressure + CO2
HbO2
HHb
Concentration
Time (s)
Mean Arterial Pressure
Pressure (mm Hg)
Arterial CO2
CO2 (mm Hg)
Arterial O2 Saturation
Saturation (%)
Scalp Blood Flux
Normalised Flux

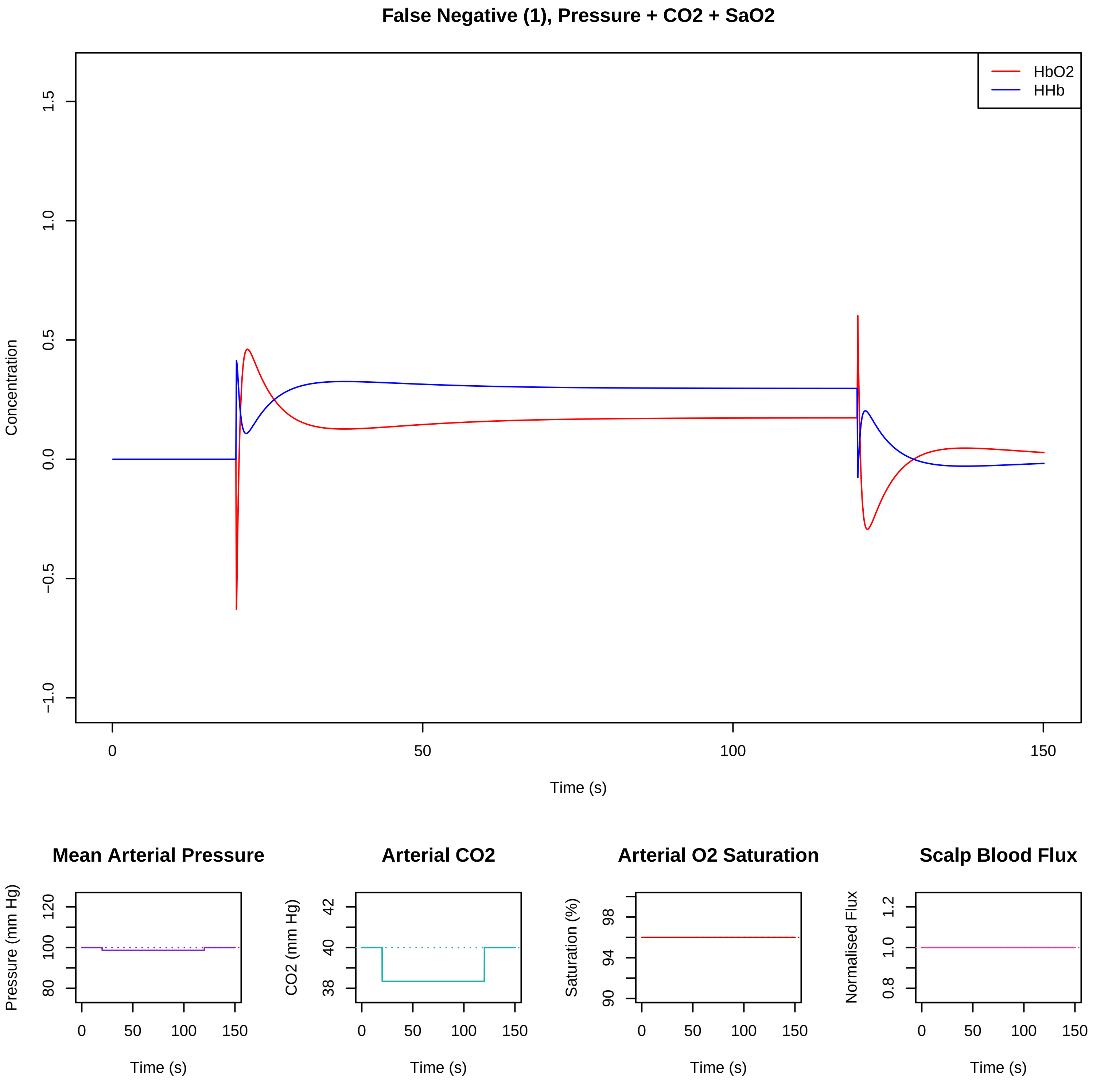
False Negative (1), Pressure + CO2 + SaO2
HbO2
HHb
Concentration
Time (s)
Mean Arterial Pressure
Pressure (mm Hg)
Arterial CO2
CO2 (mm Hg)
Arterial O2 Saturation
Saturation (%)
Scalp Blood Flux
Normalised Flux

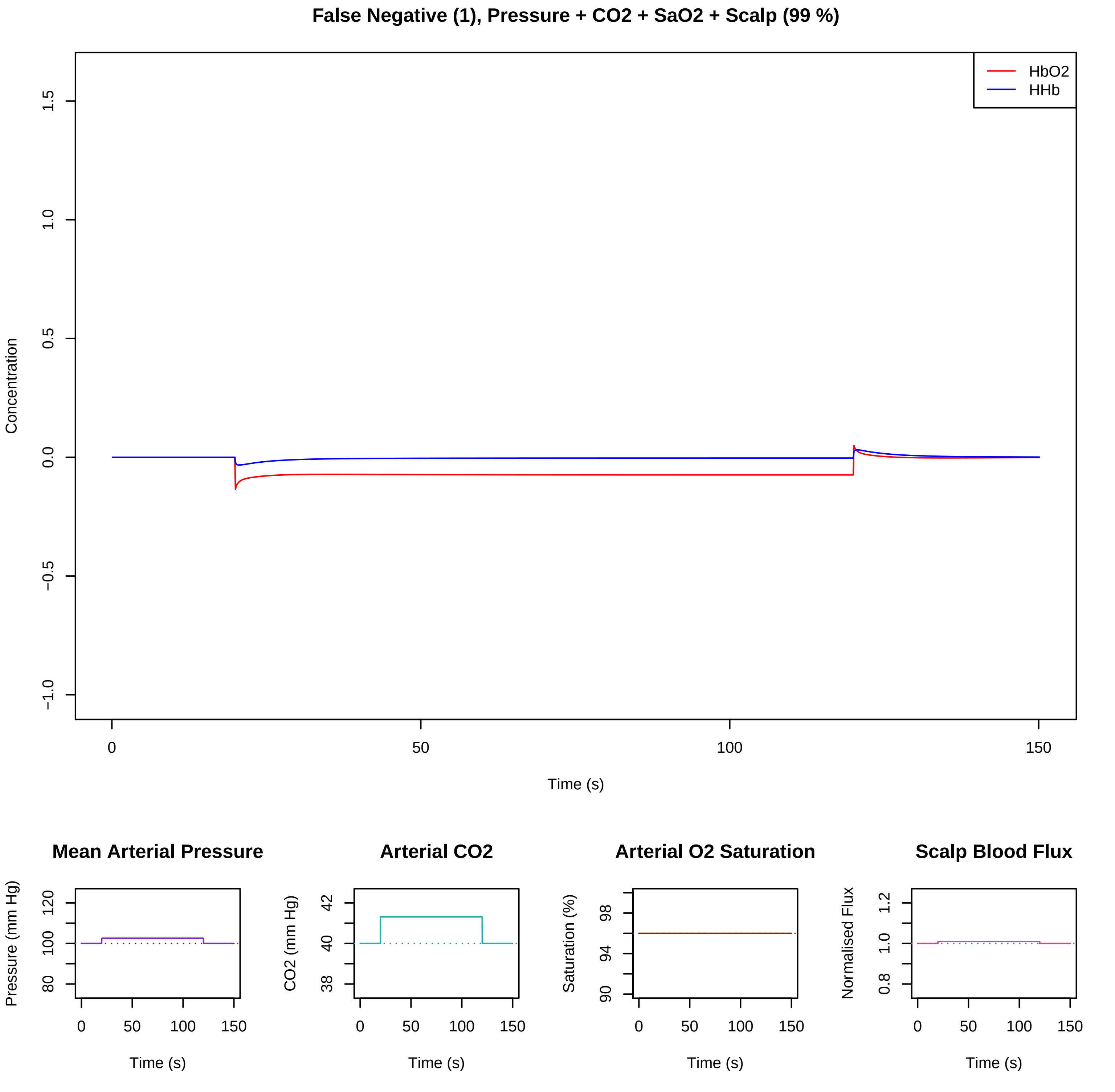

False Negative (1), Pressure + CO2 + SaO2 + Scalp (99 %)
HbO2
HHb
Concentration
1.5
1.0
0.5
0.0
−0.5
−1.0
0
50
100
150
Time (s)
Mean Arterial Pressure
Pressure (mm Hg)
80
100
120
Arterial CO2
CO2 (mm Hg)
38
40
42
Arterial O2 Saturation
Saturation (%)
90
94
98
Scalp Blood Flux
Normalised Flux
0.8
1.0
1.2

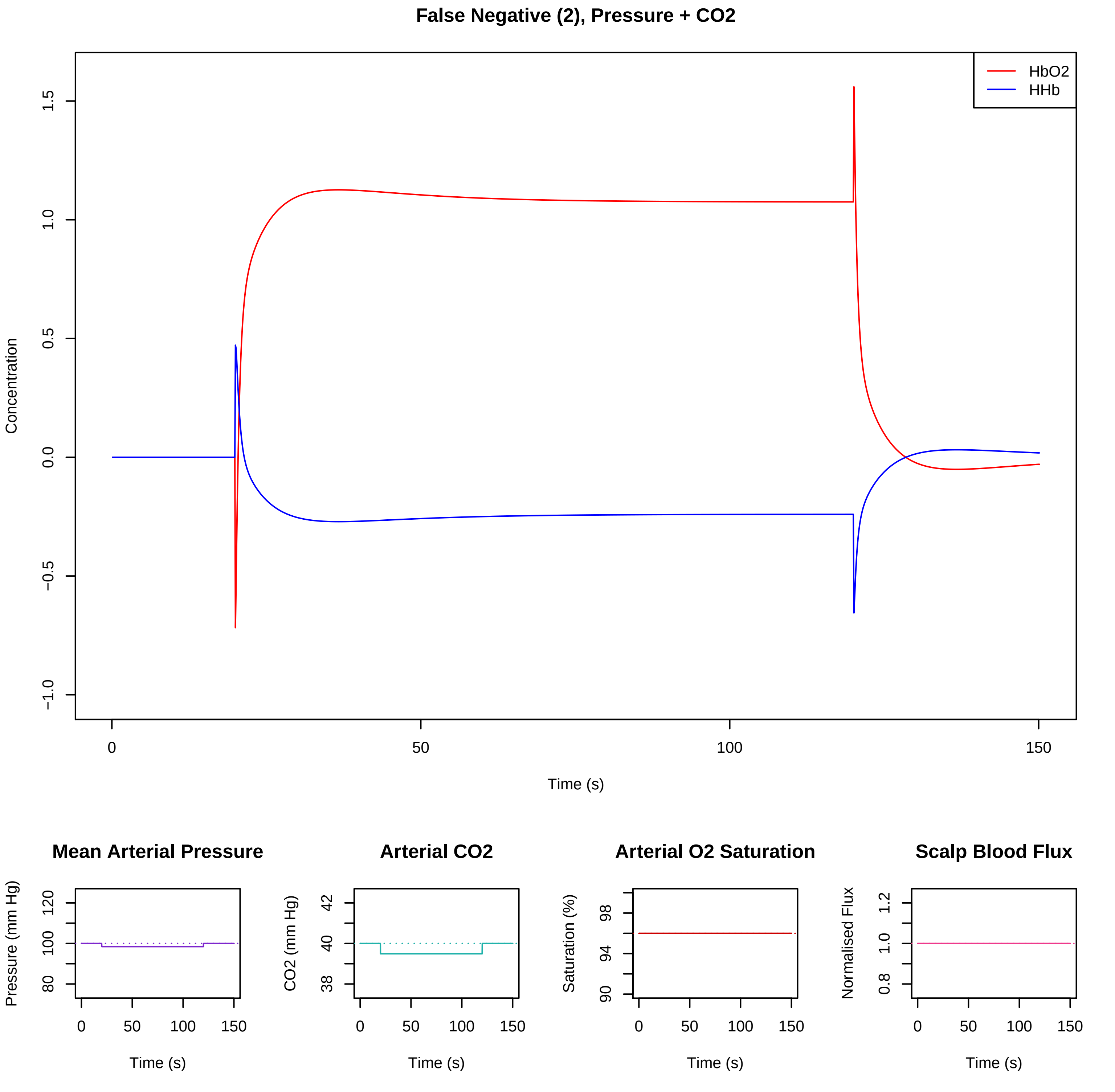

False Negative (2), Pressure + CO2
HbO2
HHb
Concentration
Time (s)
Mean Arterial Pressure
Pressure (mm Hg)
Arterial CO2
CO2 (mm Hg)
Arterial O2 Saturation
Saturation (%)
Scalp Blood Flux
Normalised Flux

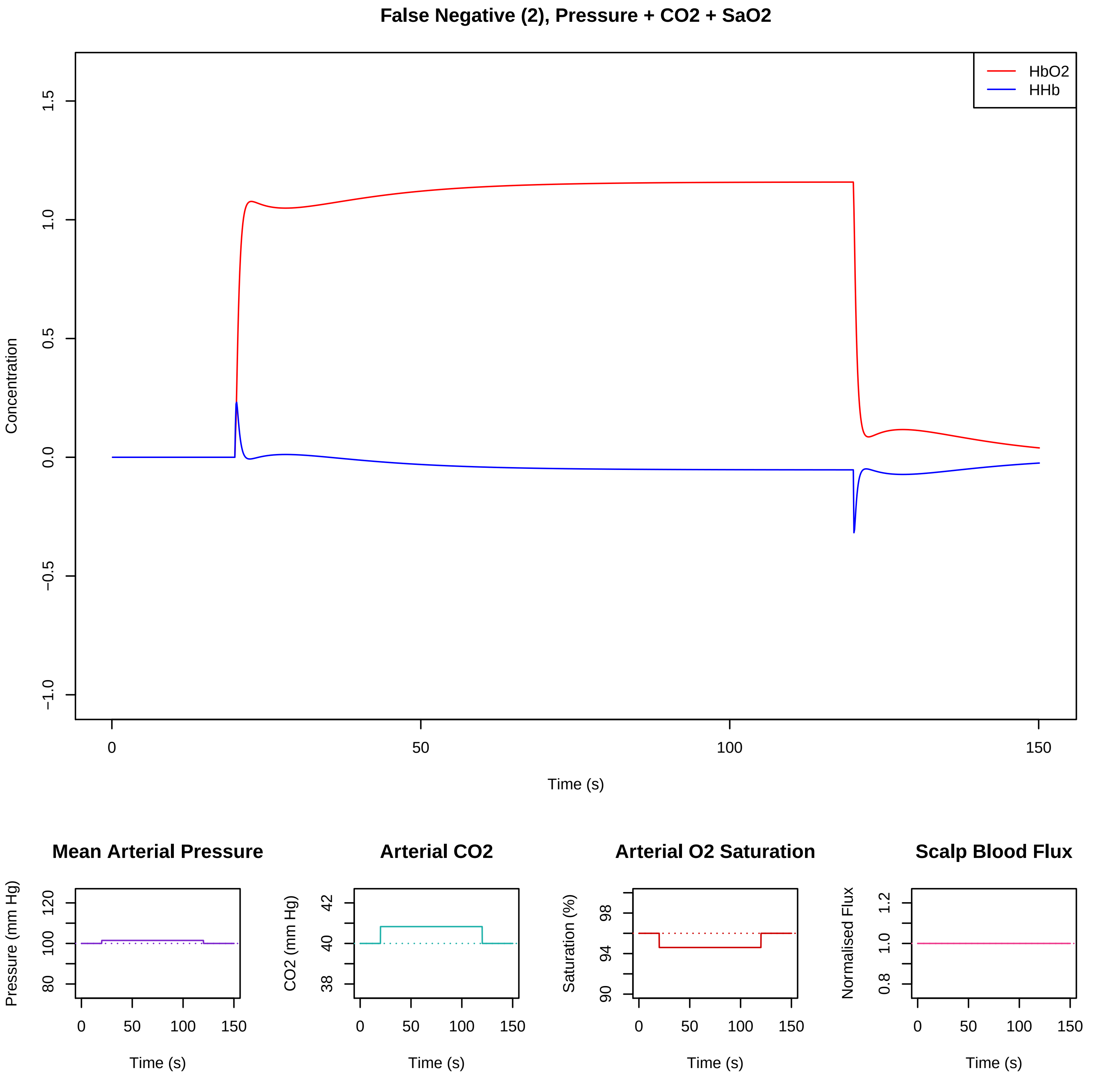
False Negative (2), Pressure + CO2 + SaO2
HbO2
HHb
Concentration
1.5
1.0
0.5
0.0
−0.5
−1.0
0
50
100
150
Time (s)
Mean Arterial Pressure
Pressure (mm Hg)
120
100
80
Arterial CO2
CO2 (mm Hg)
42
40
38
Arterial O2 Saturation
Saturation (%)
98
94
90
Scalp Blood Flux
Normalised Flux
1.2
1.0
0.8

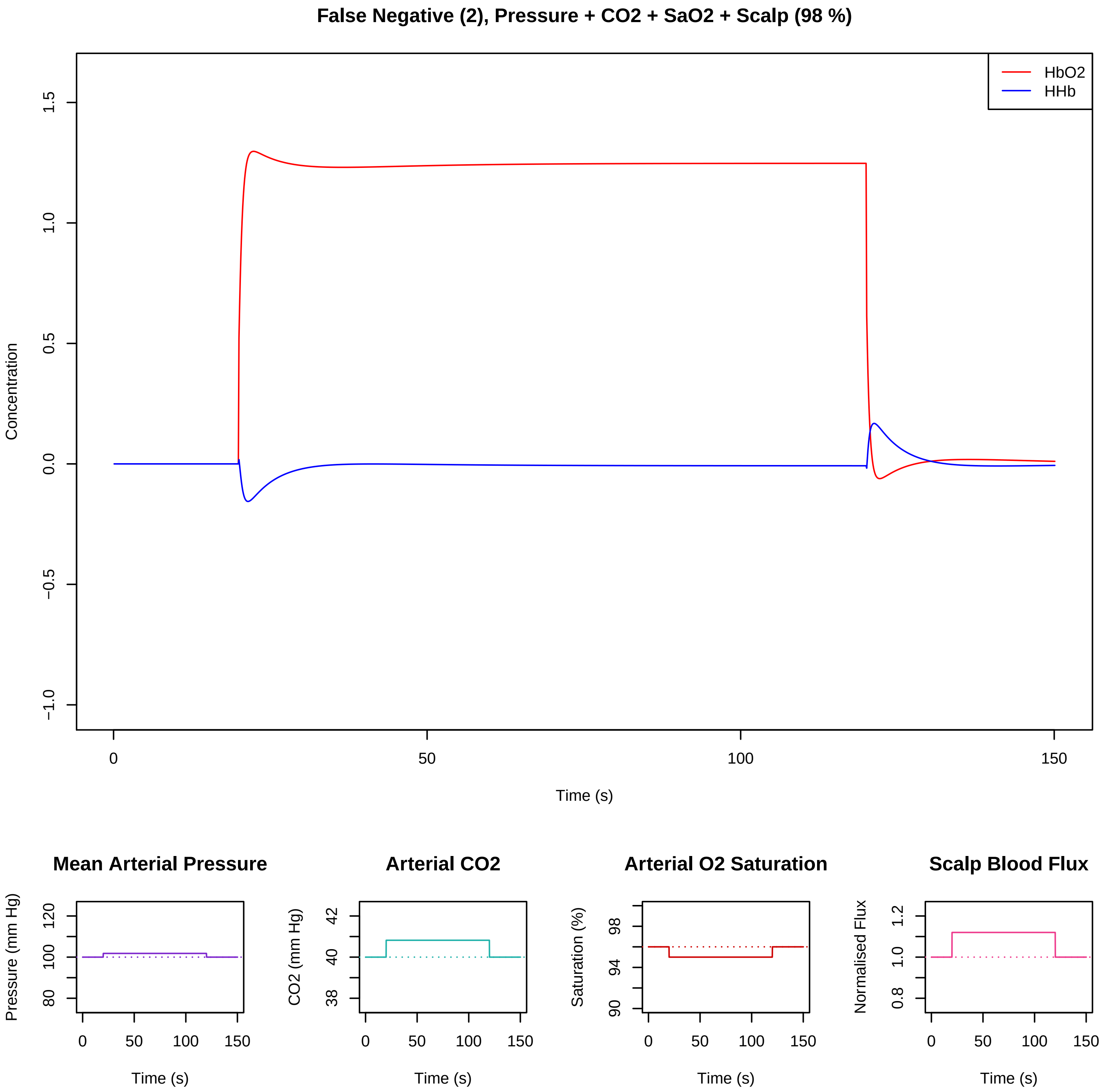
False Negative (2), Pressure + CO2 + SaO2 + Scalp (98 %)
HbO2
HHb
Concentration
1.5
1.0
0.5
0.0
−0.5
−1.0
0
50
100
150
Time (s)
Mean Arterial Pressure
Pressure (mm Hg)
120
100
80
Time (s)
Arterial CO2
CO2 (mm Hg)
42
40
38
Time (s)
Arterial O2 Saturation
Saturation (%)
98
94
90
Time (s)
Scalp Blood Flux
Normalised Flux
1.2
1.0
0.8
Time (s)

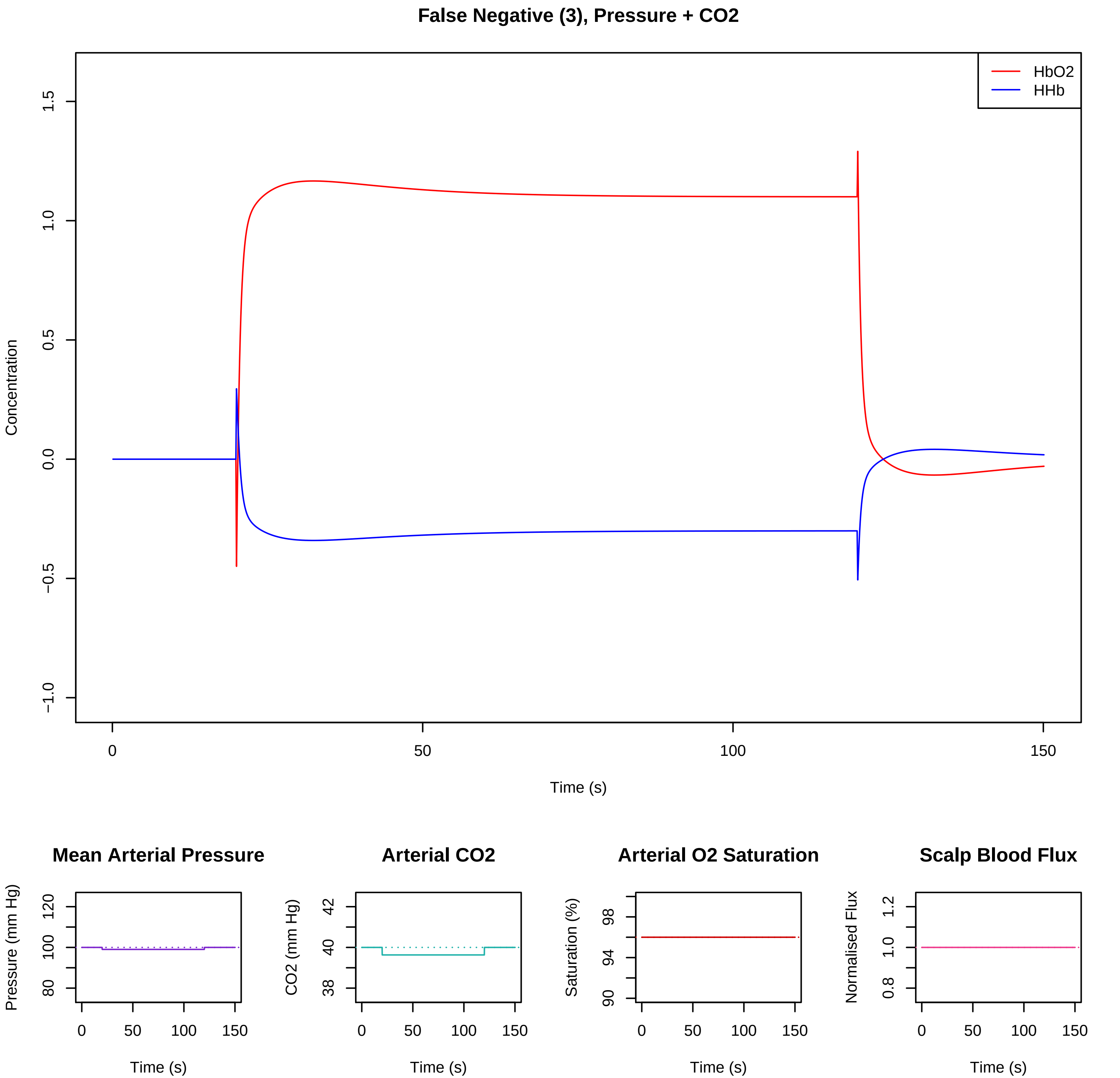

False Negative (3), Pressure + CO2
HbO2
HHb
Concentration
Time (s)
Mean Arterial Pressure
Pressure (mm Hg)
Arterial CO2
CO2 (mm Hg)
Arterial O2 Saturation
Saturation (%)
Scalp Blood Flux
Normalised Flux

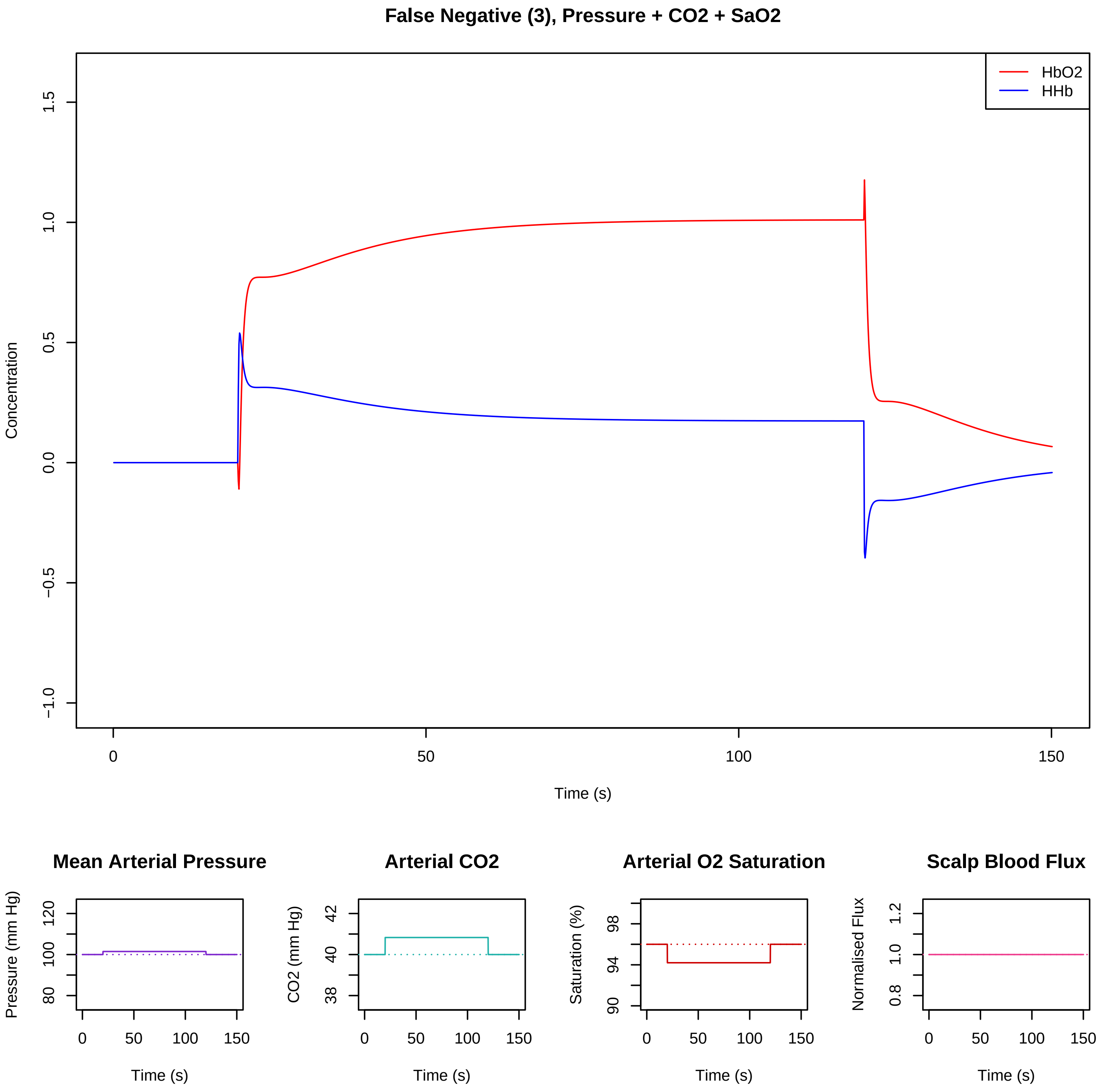

False Negative (3), Pressure + CO2 + SaO2
HbO2
HHb
Concentration
1.5
1.0
0.5
0.0
−0.5
−1.0
0
50
100
150
Time (s)
Mean Arterial Pressure
Pressure (mm Hg)
80
100
120
Arterial CO2
CO2 (mm Hg)
38
40
42
Arterial O2 Saturation
Saturation (%)
90
94
98
Scalp Blood Flux
Normalised Flux
0.8
1.0
1.2
Time (s)

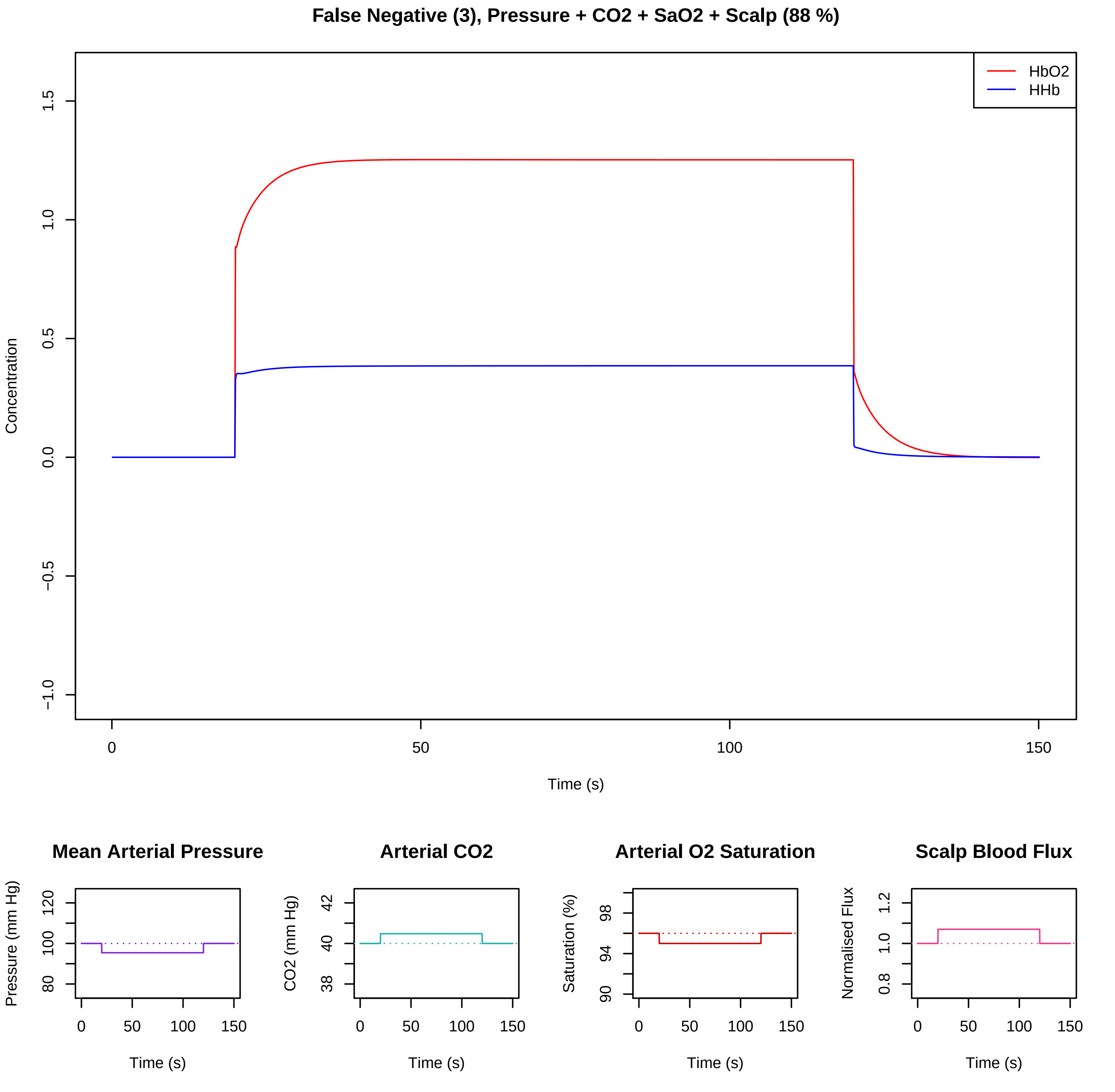
False Negative (3), Pressure + CO2 + SaO2 + Scalp (88 %)
HbO2
HHb
Concentration
1.5
1.0
0.5
0.0
−0.5
−1.0
0
50
100
150
Time (s)
Mean Arterial Pressure
Pressure (mm Hg)
80
100
120
Arterial CO2
CO2 (mm Hg)
38
40
42
Arterial O2 Saturation
Saturation (%)
90
94
98
Scalp Blood Flux
Normalised Flux
0.8
1.0
1.2
0
50
100
150
Time (s)
